# Comparative Component Analysis of Exons with Different Splicing Frequencies

**DOI:** 10.1371/journal.pone.0005387

**Published:** 2009-04-30

**Authors:** Shiqin Song, Qianli Huang, Jiaming Guo, Jesse Li-Ling, Xueping Chen, Fei Ma

**Affiliations:** 1 Department of Chemistry, University of Science and Technology of China, Hefei, China; 2 College of Life Science, Nanjing Normal University, Nanjing, China; 3 Department of Medical Genetics, China Medical University, Shenyang, China; 4 Sino-Dutch Biomedical and Information Engineering School, Northeastern University, Shenyang, China; University of Vermont, United States of America

## Abstract

Transcriptional isoforms are not just random combinations of exons. What has caused exons to be differentially spliced and whether exons with different splicing frequencies are subjected to divergent regulation by potential elements or splicing signals? Beyond the conventional classification for alternatively spliced exons (ASEs) and constitutively spliced exons (CSEs), we have classified exons from alternatively spliced human genes and their mouse orthologs (12,314 and 5,464, respectively) into four types based on their splicing frequencies. Analysis has indicated that different groups of exons presented divergent compositional and regulatory properties. Interestingly, with the decrease of splicing frequency, exons tend to have greater lengths, higher GC content, and contain more splicing elements and repetitive elements, which seem to imply that the splicing frequency is influenced by such factors. Comparison of non-alternatively spliced (NAS) mouse genes with alternatively spliced human orthologs also suggested that exons with lower splicing frequencies may be newly evolved ones which gained functions with splicing frequencies altered through the evolution. Our findings have revealed for the first time that certain factors may have critical influence on the splicing frequency, suggesting that exons with lower splicing frequencies may originate from old repetitive sequences, with splicing sites altered by mutation, gaining novel functions and become more frequently spliced.

## Introduction

The sequencing of human and mouse genomes has led to the discovery that the number of genes is not indicative of higher levels of phenotypic complexity considering the unexpectedly small number of protein coding genes [1], [2]. The number of protein coding genes is ∼25,000 in both human and mouse genomes, not significantly higher than those of the nematode genome (∼19,000) and even lower than that of the rice genome (∼40,000) [1]–[4]. Alternative splicing has been one of the important mechanisms proposed to resolve the discrepancy between gene number and organismal complexity. It has become very clear that alternative splicing not only has an extremely important role in expanding protein diversity, but also adds a regulatory dimension for the genomic expression [5]–[7].

Various mechanisms, e.g., exon skipping, intron retention, alternative 3′ and 5′ splicing sites have been identified as alternative splicing events. Studies have suggested that, in human and mouse, exon skipping is the most prevalent type and account for 38 % of conserved alternative splicing events, whilst alternative 3′ and 5′ splicing sites account for ∼18 % and ∼8 %, respectively, and intron retention is responsible for less than 3 %. The remaining ∼33 % is of complex events that include mutually exclusively alternative transcription start sites and multiple polyadenylation sites [8], [9]. Four types of signals are essential for accurate splicing, which include the 3′ and 5′ splice sites, branch site sequence located upstream of the 3′ss, polypyrimidine tract located between the 3′ss, and the branch sites [5], [10], [11]. However, accurate selection of splicing site will depend not only on the features of particular splice sites but also auxiliary regulatory motifs in the neighboring exons and introns. Recent bioinformational and experimental approaches have unraveled a large number of sequence elements that may contribute to the regulation of alternative splicing. Two types of *cis*-acting elements from exonic and intronic regions were found to have different influences on splicing by promoting recruitment of the spliceosome and exon inclusion or leading to exon skipping [12]–[16]. Based on their location and function in splicing, four major *cis*-acting regulatory elements are also recognized, including ESE (exonic splicing enhancer), ESS (exonic splicing silencer), ISE (intronic splicing enhancer) and ISS (intronic splicing silencer). Many ESE elements act as binding sites for a family of proteins known as SR (serine/arginine-rich) proteins, and participate in both alternative and constitutive splicing [12], [17], [18].

Previous studies have also shown that ASEs possess several features that distinguish them from CSEs, such as weaker signals at alternative splicing sites, shorter lengths, higher level of sequence conservation, longer flanking intronic sequences, involvement of repetitive elements on exonization, and greater frequency for skipping exons to preserve the reading frame. [8], [9], [19]–[27]. At present, most studies have focused on the difference between ASEs and CSEs. However, transcriptional isoforms may be not just random combinations. Why? And why do some exons draw out from the aggregation more frequently and others do not? In fact, very little is known about the precise mechanism behind exons that exhibit different splicing frequencies.

Some previous studies have shown that many elements influenced the regulation of ASEs' splicing. For example, Zhang and coauthor had reported the influences of ESE elements on the splicing of exons [16]. Zheng and coauthor had found that distinct sequence and structural features between alternative splicing and constitutive splicing, including exon length and repetitive elements, have very important effect on the splicing of ASEs [26]. Moreover, the GC-content has also been systematically studied as an important component of exon sequences [28], [29]. To elucidate why different exons exhibit different component property within their transcripts, we have systematically investigated features of different exon groups with different splicing frequencies (G1 group, G2 group, G3 group, G4 group, see *Materials and Methods* for details) from alternatively spliced human genes and their mouse orthologs, including exon length, GC content, ESE and repetitive elements.

## Results

### Exon length distribution of different exonic groups

**Figure 1:**
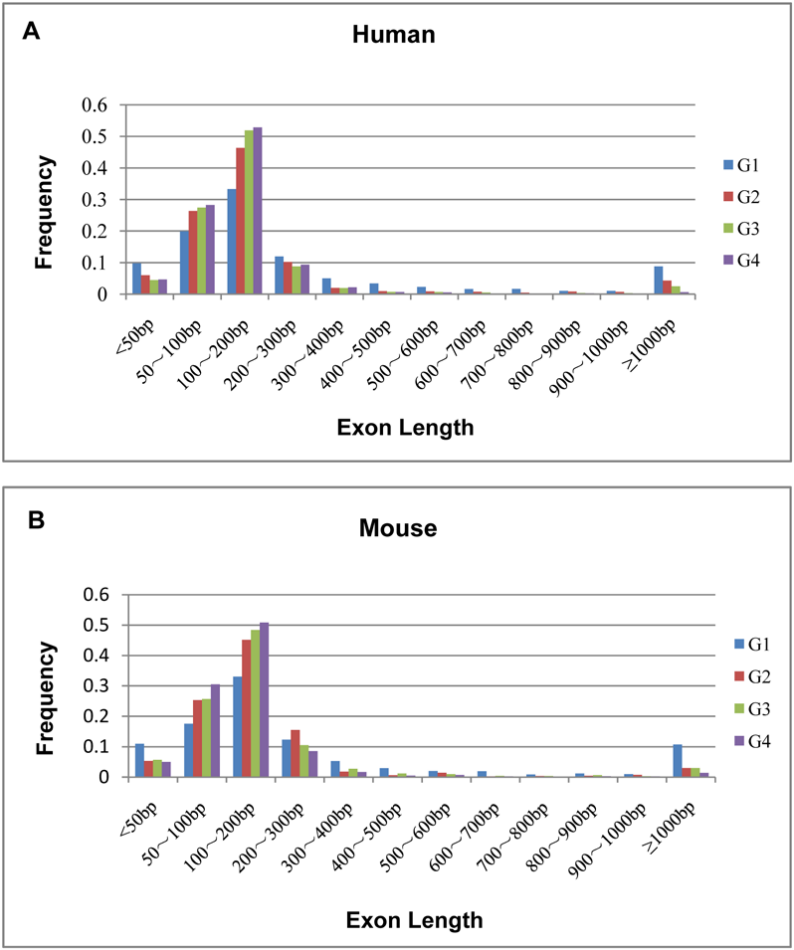
Distribution of exon lengths of four groups for alternatively spliced human genes (A) and mouse orthologs (B).

Previous studies have suggested that exon length may influence the selection of splice site. In present study, we have systematically investigated exon length distribution in the four groups of exons extracted from selected human and mouse genes (Table 1 and Figure 1). As shown in Table 1, the average lengths of the four exonic groups for alternatively spliced human genes were, respectively, 369.36 (G1), 249.61 (G2), 191.18 (G3) and 154.34 nt (G4), whereas the average exonic lengths for their mouse orthologs were, respectively, 428.27 (G1), 226.41 (G2), 218.52 (G3) and 159.42 nt (G4). Therefore, the average lengths of exons have decreased significantly along with the increase of splicing frequencies in both species. Statistically, significant differences existed between the average lengths of four exonic groups in both human (F = 128.24, *p*<0.0001) and mouse (F = 64.02, *p*<0.0001). Furthermore, G1 group seemed to contain exons with length greater than 300 nt for a remarkably higher proportion (human: 25.01 %; mouse: 26.08 %) than G2 (human: 11.04 %; mouse: 8.72 %), G3 (human: 7.39 %; mouse: 9.71 %), and G4 group (human: 4.92 %; mouse: 5.05 %), suggesting that shorter exons have a tendency to be more frequently spliced. Furthermore, as shown in Figure 1, the four types of exons exhibited very similar length distribution in human and mouse, which also implied a functional significance of such properties.

**Table 1:** Average lengths of exons spliced with different frequencies in human and mouse.

	Number of Exon				Minimum Length				Maximum Length				Average Length (±SD)			
	G1	G2	G3	G4	G1	G2	G3	G4	G1	G2	G3	G4	G1	G2	G3	G4
**Human**	5477	1730	1935	3172	11	11	15	12	11710	11846	5073	4381	369.36 (685.5)	249.61 (570.92)	191.18 (341.08)	154.34 (205.11)
**Mouse**	1971	826	1359	1308	11	12	11	12	15963	5527	5970	5398	428.27 (866.08)	226.41 (438.71)	218.52 (445.97)	159.42 (246.01)

### Distinct GC properties of the four exonic groups

Discrepancies in GC content among the four exonic groups were further examined. For exons from the human gene dataset, the GC values varied between 53.37 %±11.61 % (9.5 % ∼ 90 %) (G1 group), 52.06 %±10.24 % (26.9 % ∼ 87.5 %) (G2 group), 51.65 %±10.05 % (25.8 % ∼ 88.7 %) (G3 group), and 50.56 %±10.07 % (26.8 % ∼ 86.5 %) (G4 group). For the mouse gene dataset, the GC values varied between 52.72 %±10 % (0 to 85.2 %) (G1 group), 51.71 %±8.73 % (22.2 % ∼ 88.9 %) (G2 group), 51.86 %±8.46 % (25 % ∼ 83.3 %) (G3 group), and 53.19 %±7.82 % (26.4 % ∼ 81.4 %) (G4 group). Apparently, the GC content has varied substantially among the four groups in both human and mouse. Particularly, G1 exons showed relatively higher heterogeneity than other groups in both species.

An F-test was carried out to assess the differences in GC contents between the four groups of exons. For human genes, highly significant differences were found between G1 and G2 (F = 1.29; *p*<0.0001), G1 and G3 (F = 1.33; *p*<0.0001), or G1 and G4 exons (F = 1.33; *p*<0.0001), while none was found between G2 and G3 (F = 1.04; *p* = 0.4254), G2 and G4 (F = 1.03; *p* = 0.4285), or G3 and G4 exons (F = 1.00; *p* = 0.9243). For mouse genes, significant differences were found between G1 and G2 (F = 1.31; *p*<0.0001), G1 and G3 (F = 1.39; *p*<0.0001), or G1 and G4 exons (F = 1.63; *p*<0.0001), while nonsignificant difference was found between G2 and G3 (F = 1.06; *p* = 0.3238). However, compared to human, significant differences were also found between G2 and G4 (F = 1.24; *p* = 0.0004), or G3 and G4 exons (F = 1.17; *p* = 0.0040) in mouse.

**Figure 2:**
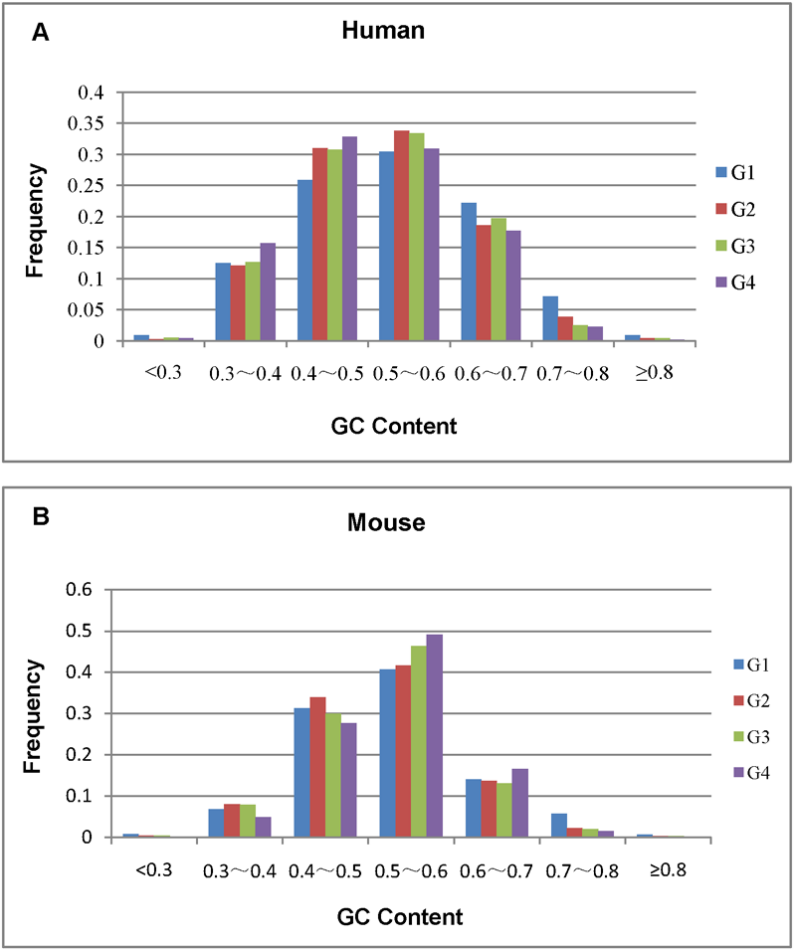
Distribution of GC content of the four types of exons for alternatively spliced human genes (A) and mouse orthologs (B).

Notably, the GC content of G1 exons is somewhat higher than those of other types. When extreme GC content of 60 % or more was considered, in human and mouse, respectively, 30.25 %, 20.35 % of the G1, 22.89 %, 16.10 % of the G2, 22.68 %, 15.31 % of the G3, and 20.11 %, 18.20 % of the G4 exons may fit in this category (Figure 2). This seems to suggest that exons of lower GC content tend to be more frequently spliced in both species.

### Influence of ESE elements on different exonic groups

**Figure 3:**
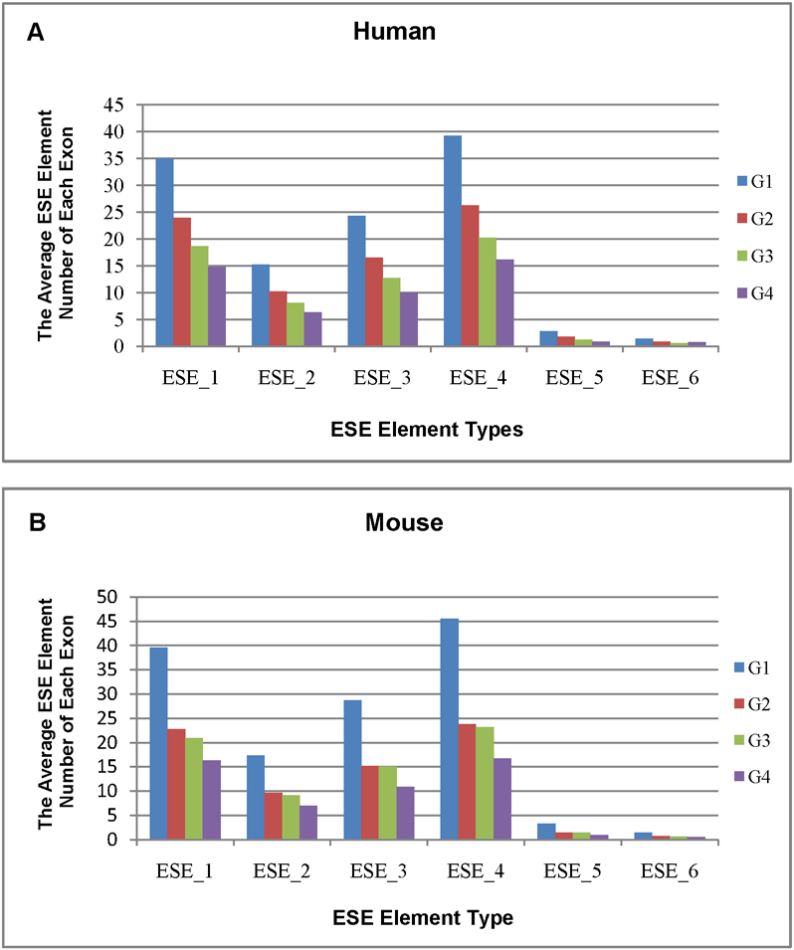
Average numbers of ESE elements of different exonic groups for alternatively spliced human genes (A) and mouse homologs (B).

Average ESE element usage in the four exonic groups was summarized in Figure 3 (Table S1 and S2). As shown, the four groups of exons exhibited a very similar preference for ESE usage in both human and mouse, with the order in the four groups being ESE_4>ESE_1>ESE_3>ESE_2>ESE_5>ESE_6. It is also interesting to note that ESE elements of similar usage among the four groups have been the preferred ones, such as ESE_4, ESE_1 and ESE_3, which seemed in keeping with previous reports that ESE elements are highly conserved between alternatively spliced human genes and mouse orthologs [8], [21], [25], [30]. This seems to imply that, to maintain effective exon splicing, such organisms have endured similar selective pressure during the evolution, which in turn has resulted in a similar tendency for ESE elements usage across different types of exons.

As shown in Figure 3, the average numbers of six types of ESE elements for each group of exons showed a significant decrease along with the increase in splicing frequency in both human and mouse, suggesting that exons with fewer ESE elements tend to be more frequently spliced. Considering the enhancer role of ESE, above results appeared to be paradoxical. However, the decision of splicing is complicated and may be influenced by compensatory factors. For instance, weak splice signals in splicing sites may be a complement factor in such case. Hence, the numbers of ESE elements in exons may also have an important influence on splicing frequencies.

As shown in Table 2, significant differences were also found between alternatively spliced human genes and mouse orthologs grouped by exonic and ESE types (Table 2). This suggested that different splicing frequencies are associated with not only exon type, but also ESE category in the two species. ESE elements in exons may also influence the splicing frequencies.

**Table 2:** Statistical significance for differences between alternatively spliced human genes and mouse orthologs grouped by exonic and ESE types.

	Human		Mouse	
	Chi-Square	Pr>ChiSq	Chi-Square	Pr>ChiSq
**ESE**	149621.8	<.0001	37258.42	<.0001
**Exon group**	191658.2	<.0001	120152	<.0001
ESE*Exon group	5488.71	<.0001	2952.42	<.0001

### Repetitive elements found in different groups of exons

Repetitive elements from different exonic groups of human and mouse genes were outlined in Table 3. The proportions of exons with repeat element in human and mouse were, respectively, 14.39 % and 12.99 % (G1), 5.67 % and 4.84 % (G2), 3.72 % and 4.41 % (G3), and 2.05 % and 2.12 % (G4). Clearly, repetitive elements in G1 type exons are more abundant than other groups in both species, and there was a downtrend for the proportions of exons with repetitive element along with the increase in splicing frequencies, suggesting that frequently spliced exons tend to avoid from holding repetitive elements than infrequently spliced ones.

**Table 3:** Proportion of exons that contain repetitive elements in human and orthologous mouse genes.

	Number of exons with repetitive elements				Total number of exons				Proportion of exons with repetitive elements (%)			
	G1	G2	G3	G4	G1	G2	G3	G4	G1	G2	G3	G4
**Human**	788	98	72	65	5477	1730	1935	3172	14.39	5.67	3.72	2.05
**Mouse**	256	40	60	22	1971	826	1359	1308	12.99	4.84	4.42	2.12

The number of repetitive elements in the four exonic groups was further analyzed. As shown in Table 4, each group had contained different numbers of repetitive elements. Particularly, ASEs with lower splicing frequencies (G1 exons) contained more repetitive elements in both human and mouse. This seems to imply that transposable elements, including short interspersed nuclear elements (SINEs), long interspersed nuclear elements (LINEs), long terminal repeats (LTRs), DNA transposons (DNAs), and simple repetitive sequences, e.g., Low complexity repeats (LCRs) and Simple repeats are preferentially associated with exons with lower splicing frequencies. That said exons containing more repetitive elements tend to be spliced at lower frequencies.

**Table 4:** Repetitive elements found in different groups of exons from human and mouse.

Species	Exonic group							
	G1		G2		G3		G4	
	Human	Mouse	Human	Mouse	Human	Mouse	Human	Mouse
**DNA type**								
DNA	2	0	0	0	0	0	0	0
DNA/AcHobo	4	0	0	0	0	0	0	0
DNA/hAT	2	0	1	0	0	0	0	0
DNA/Mariner	2	1	0	0	0	1	0	0
DNA/MER1_type	55	5	4	0	3	1	0	0
DNA/MER2_type	15	1	0	0	0	0	0	0
DNA/Tc2	4	0	0	0	0	0	0	0
DNA/TcMar	2	0	0	1	2	0	0	0
DNA/TcMar?	1	0	0	0	0	0	0	0
DNA/Tigger	1	0	0	0	0	0	0	0
DNA/Tip100	1	0	0	0	0	0	0	0
**LINE type**								
LINE/CR1	6	2	1	0	0	0	1	0
LINE/L1	67	13	5	5	2	3	0	0
LINE/L2	50	5	8	1	4	0	4	0
LINE/RTE	4	0	0	0	0	0	0	0
**LTR type**								
LTR/ERV1	17	1	1	0	2	1	1	0
LTR/ERVK	4	8	0	1	0	0	0	0
LTR/ERVL	12	1	1	0	2	0	0	0
LTR/MaLR	31	12	1	0	0	7	0	0
**SINE type**								
SINE	3	0	0	0	0	0	0	0
SINE/Alu	241	48	18	2	14	12	7	2
SINE/MIR	105	9	11	1	3	1	1	0
SINE/tRNA	2	0	0	0	0	0	0	0
SINE/B2	0	13	0	0	0	3	0	0
SINE/B4	0	20	0	1	0	5	0	0
SINE/ID	0	2	0	0	0	5	0	0
**Other types**								
scRNA	0	3	0	0	0	2	0	0
srpRNA	2	0	0	0	0	0	0	0
snRNA	0	1	0	0	0	0	0	0
tRNA	1	0	0	0	0	0	0	0
RNA	1	0	0	0	0	0	0	0
Other	2	4	0	0	0	0	0	0
LCRs	397	156	51	30	61	36	38	9
Simple_repeat	264	181	52	24	29	41	36	15

### Comparison between NAS mouse genes and alternatively spliced human orthologs

To explore the evolution of ASEs, we had further explored connection between NAS mouse genes and their alternatively spliced human orthologs. Features of exons from the two groups of genes were also systematically surveyed.

#### Exon length

With median values being 231.63 and 275.05 nt, respectively, the average lengths of exons differed between mouse NAS genes and alternatively spliced human orthologs (Table 5). Notably, the median length of G1 exons had measured 359.54 nt, much higher than those of G2 (224.29 nt), G3 (185.43 nt) and G4 exons (157.18 nt) (Table 5). In addition, exon numbers in human genes (2,338) were significantly higher than those of mouse genes (1,633). Particularly, exon number in G1 group (1,197) was higher than other groups. These seemed to suggest a substantial increase in both exonic length and number during the evolution from constitutive splicing to alternative splicing, and that ASEs with lower splicing frequencies are the predominant form during evolution. Moreover, the large variation in exon lengths between mouse NAS genes and alternatively spliced human orthologs, as shown in Table 5, may also reflect a combined influence on splicing regulation for different types of exons.

**Table 5:** Exonic lengths for mouse NAS genes and alternatively spliced human orthologs.

	Number of Exons	Minimum length	Maximum length	Average length (±SD)
**NAS mouse**	1633	13	6453	231.63 (430.14)
**AS human**	2338	13	9453	275.05 (502.39)
**human G1**	1197	13	9453	359.54 (627.9)
**human G2**	353	16	5454	224.29 (415.9 )
**human G3**	342	23	3050	185.43 (287.4)
**human G4**	446	23	1793	157.18 (156.27)

#### GC content

As shown in Table 6, highly significant differences also existed in the average GC content of exons between mouse NAS genes and alternatively spliced human orthologs (F = 1.65, *p*<0.0001), but none was found between mouse NAS and human G4 exons (F = 1.13, *p*<0.1102). Particularly, the average GC content of G1, G2 and G3 exons are higher than that of human G4 group and mouse NAS genes, suggesting that the average GC contents of ASEs has increased significantly during evolution, confirming that GC content probably has an important influence on splicing frequency in alternatively spliced human genes.

**Table 6:** GC contents for mouse NAS genes and alternatively spliced human orthologs.

Species	Number of Exons	Minimum GC	Maximum GC	Average GC(±SD)
**NAS mouse**	1633	25 %	80.8 %	51.48 % (8.64 %)
**AS human**	2338	16.7 %	90 %	53.05 % (11.9 %)
**human G1**	1197	16.7 %	90 %	53.87 % (11.99 %)
**human G2**	353	28 %	80 %	53.14 % (10.23 %)
**human G3**	342	28.1 %	80 %	53.41 % (10.5 %)
**human G4**	446	27.9 %	82.4 %	50.49 % (9.16 %)

#### ESE element

Averaged ESE element numbers per exon in mouse NAS genes and alternatively spliced human orthologs were outlined in Table 7. As shown, ESE element numbers per exon in alternatively spliced human genes were higher than those for mouse NAS genes, suggesting that ESE element may play important roles in the evolution from constitutive splicing to alternative splicing. Analysis has also found the averaged ESE element in human G1 exons (115.28) to be significantly higher than those of G2 (71.63), G3 (62.13) and G4 exons (49.76), and that the average values in human G3 and G4 exons were also much lower than that of mouse NAS genes. This seems to suggest that ASEs with lower splicing frequencies have gained more ESE elements during the evolution. Taking together, ESE elements in exons have very an important effect on the splicing frequency in humans.

**Table 7:** Average numbers of ESE elements per exons in NAS mouse genes and human orthologs.

ESE Type	Exonic group					
	Mouse NAS	Human AS	human G1	human G2	human G3	human G4
**ESE_1**	21.70	26.88	34.65	21.80	20.13	15.22
**ESE_2**	9.84	11.76	15.23	9.50	8.58	6.65
**ESE_3**	15.91	18.01	23.46	14.82	12.48	10.18
**ESE_4**	24.64	28.68	37.61	23.08	19.10	16.48
**ESE_5**	1.58	2.10	2.92	1.67	1.32	0.83
**ESE_6**	0.75	0.99	1.41	0.76	0.512	0.40
**Total ESE**	74.42	88.42	115.28	71.63	62.13	49.76

#### Repetitive element

To explore the potential roles of repetitive elements in the evolution from constitutive splicing to alternative splicing, we have performed a wide analysis on SINEs, LINEs, LTRs, DNAs, LCRs and simple repeats (Table 8). As shown in Table 8, the total number of repetitive element within exons of alternatively spliced human genes (365) are two times higher than that of mouse NAS genes (164), and that SINEs, LINEs, DNAs, LCRs, simple repeat are preferentially associated with human ASEs, with SINEs and LCRs types being the major categories. Furthermore, the numbers of repetitive elements increased primarily in human G1 exons, suggesting that such elements may contribute to the creation of novel ASEs with the lowest splicing frequencies.

**Table 8:** Total numbers of repetitive elements in NAS mouse genes and alternatively spliced human orthologs.

Repeats Type	Mouse NAS	Human AS	Human G1	Human G2	Human G3	Human G4
**DNA Type**	6	45	42	3	0	0
**LINE Type**	9	41	38	2	1	0
**SINE Type**	51	99	88	5	5	1
**LTR Type**	5	22	22	0	0	0
**LCRs**	47	91	60	9	14	8
**Simple_repeat**	46	67	52	5	8	2
**Total repeats**	164	365	302	24	28	11

### The relationships between splicing frequency and splicing pattern

From above analyses we found that exons with different splicing frequency have actually exhibited distinct components features. However, whether these different features are caused by different splicing pattern of exons? In the present work, we selected five main splicing patterns for further study (see *Materials and Methods* for details). Statistic results indicated that there are no significant statistical differences between the proportion value of each exon group (see *Materials and Methods* for details) for each splicing pattern in both alternatively spliced human and mouse (F = 0.04, *p* = 0.9904 and F = 0.01, *p* = 0.9980 for human and mouse, respectively). Our results displayed that there is no relationship between splicing frequency and splicing pattern in both human and mouse genes, suggesting that different exons undergo different splicing frequency may come from these instinct component features of exons.

## Discussion

Our analysis has indicated that exons with the lowest splicing frequencies are in average significantly longer in alternatively spliced human genes and their mouse orthologs. The two groups of genes also exhibited similar exonic length distribution across the four exonic groups (Figure 1). This, on one hand, has suggested conservation of functional properties between human and mouse and, on the other hand, indicated that exon length may be an important index for splicing frequency of exons in both human and mouse. Our results may provide important complement to previous discoveries that ASEs tend to be shorter than CSEs in alternatively spliced human genes and mouse orthologos [8], [19]–[23]. As shown in present study, exons with the lowest splicing frequencies are significantly longer than CSEs or other types of exons (Table 1). The possible explanation for this may lay in the difference in dataset selection. Previous studies have primarily focused on different models of alternative splicing, which mainly investigated ASEs of different splicing patterns, particularly skipped exons, while our work just has focused on exons with different splicing frequencies.

Previous studies have demonstrated that, at least for human and mouse, various types of exons may be different in length. For instance, skipped exons are significantly shorter, whereas retained introns are significantly longer than CSEs [20], [26], [31], [32]. Zheng *et al.* also found that, compared with CSEs, constitutive portion of alternative acceptor and alternative donor exons are similar in length, but their alternative portions are shorter. Based on above findings, it seems deducible that longer ASEs in our dataset may have included retained introns, alternative acceptor and/or donor exons. This may in part explain that exons with the lower splicing frequencies for being longer in our studied alternatively spliced human and orthologous mouse genes.

As described above, the average GC content are divergent among different exonic groups in both alternatively spliced human genes and mouse homologs (Figure 2). Exons of lower GC content tend to be more frequently spliced in both species. This may not be incidental. In present study, the average length of exons has significantly decreased along with the increasing of splicing frequency in human and mouse. That said, frequently spliced exons tend to be shorter and lower in GC content. This seems to be sustained by two previous studies [28], [29]. Oliver and Marin had predicted that exon lengths should increase with the GC content and that the expected length of reading frames in random sequences is thus a function of GC content. The authors presented theoretical arguments and empirical evidence that the longest eukaryotic exons are GC-rich ones. This, on one hand, has indicated that the differential expansion of coding sequences may be constrained by compositional heterogeneity pervading most genomes. On the other hand, it also suggested dependence for divergent sequence feature on differential exon splicing.

Previous studies have predicted the ASEs to have different frequencies of ESE and ESS elements compared with CSEs. However, most of such studies had focused on skipping-exons [33], [34]. In present study, we determined the predicted ESEs for each exonic group regardless of splicing models with the aim to capture regulatory properties of differently spliced exons which with different splicing frequencies. As shown, despite the different amounts of ESEs in differently spliced exonic groups, contributions of ESEs to the splicing of exons are not significantly different (Figure 3). It may therefore be concluded that different amounts of regulatory elements may be the dynamism that drove exons to be spliced with different frequencies. Furthermore, along with the increased splicing frequency, the average numbers of ESE that required for splicing have gradually decreased in both human and mouse (Figure 3). This seems to be consistent with previous report that ASEs contain more potential regulatory sequences than CSEs do [20]. Notably, exons with the lowest splicing frequency tend to contain the largest amount of ESE. Taking together, these seem in agreement with previous reports that minor-form ASEs require more regulatory signals than major-form ASEs and that their splicing may be more complicated regulated [35]. Furthermore, our results have implied that information such as ESE diverges not only between ASEs and CSEs, but also among exons with different splicing frequencies. The significant difference in the ESE elements usage also seemed to imply biological changes as the result of evolution. The patterns of ESE elements usage may therefore reflect mechanisms crucial for understanding of the evolution and origin of ASEs.

The similar tendency in ESE usage in human and mouse also seem to have confirmed the significance of regulatory elements in alternative splicing and conserved regulation by such elements [8], [21], [25], [30], [36]. It has also indicated that alternatively spliced human and mouse genes have endured similar selective pressure during the evolution. On the whole, exons with the lower splicing frequencies may require more ESE elements for accurate splicing.

As revealed by our analysis, different groups of exons tend to possess different amounts of repetitive elements. Particularly, ASEs with lower splicing frequency contained more repetitive elements (Table 4). In addition, transposable elements including SINEs, LINEs, LTRs, and DNAs are preferentially associated with ASEs, which also coined with previous reports that such elements play a more specific role in the evolution of ASEs than other types of repetitive elements [26]. Preferential possession of repetitive element by infrequently spliced exons may also find support from previous discoveries that transposable elements inserted into intronic regions can evolve into exons through exonization [37]–[45]. Some researchers have suggested that newly created exons had firstly appeared as minor-form isoforms and gradually gain functions through the evolution [38], [41], [44], [45]. In our datasets, the proportions of exons containing repetitive elements in the four groups decreased from G1 to G4. In particular, exons with the lower splicing frequencies tend to contain more repetitive elements, which seem to indicate the former to be new created exons that underwent alternative splicing recently.

Comparing mouse NAS genes with orthologous alternatively spliced human genes has revealed that certain properties of exons, such as length, exon number, GC content, ESE and repetitive elements have been altered during the evolution. Particularly, ASEs with the lower splicing frequencies showed more obviously changes. These further suggested that ASEs with the lowest splicing frequency have been a main evolutionary product from CSEs. A recent research has indicated that evolution from CSEs to ASEs usually combined with relaxation of 5′ splicing site and fixation of exonic splicing regulatory sequences [46]. Therefore, as revealed by present study, lower splicing frequency, suddenly increased number, greater exon lengths, higher GC content, more ESE and repetitive elements may all characterize ASEs evolved from CSEs. This seems to be sustained by existing theories that minor-form exons are recently created [38], [41], [44], [45]. Moreover, increased repetitive elements (transposable elements in particular) in human G1 exons also seem to imply that such elements have contributed to the origin of alternative splicing [26]. Recent researchers have found evidence that many new ASEs have evolved from repetitive elements inserted into intronic regions [40], [41], [45]. Therefore, it may be concluded that repetitive elements have important contributions to the creation of ASEs during the evolution, and that exons with the largest amount of repetitive elements are initially driven to be spliced with the lowest splicing frequency, and became more frequently spliced through the evolution.

Some researchers have systematically summarized the evolution of alternative splicing and exons [7], [47]–[51]. Human-mouse comparisons have revealed that alternative splicing is often associated with accelerated rate of exon creation and/or loss in particular species [38], [52], [53]. Recent studies have described two mechanisms for exon creation, which included exon shuffling [54], [55] and exonization of intronic sequences [37], [39]–[41], [43], [45]. It has been suggested that highly repeated sequences are the most important source of new exons in both human and mouse [40]. Particularly, Alu repetitive elements can be exonized through a small number of mutations to create new alternative splicing sites [43], [56]. More than 5 % of alternatively spliced internal exons in the human genome have been shown to derive from Alu elements [40], [42], [57]. As proposed by Zhang and Chasin , 40 % of new human exons are alternatively spliced, most of which are cassette exons with low inclusion rates, and the majority (90 %) of new cassette exons resemble genomic interspersed repetitive sequences [41]. Studies have also suggested that new exons appeared initially as minor splicing isoforms, gradually gained functions with time, and became constitutive exons correlated with mutations that creating stronger splice sites [38], [41], [44], [45]. This may in part account for the fact that exons with more ESE are still infrequently spliced.

Taking together, we proposed that exons with lower splicing frequencies maybe newly created ASEs, which exhibit greater lengths and higher GC content, and contain more ESE and repetitive elements. Such exons may have originated from old repeated sequences, with splicing sites altered by mutation, and gained functions with time, and eventually became more frequently spliced. In this study, we have explored the potential components embedded in exons classified according to splicing frequencies. According to our analyses, differently spliced exons seem to exhibit significantly different properties, which in part may give rise to various splicing frequencies. As suggested by our results, the splicing frequency may be an intrinsic property divergently regulated by features such as ESEs, exon length, GC-content and repetitive elements. Here, we have addressed some conclusions of the probability that different exons with different splicing frequencies actually exhibit different component features in alternatively spliced human and mouse genes. Moreover, by the analysis of the relationship between splicing frequency and splicing pattern, we may draw a conclusion that different exons have no statistical differences between splicing frequency and splicing pattern in both human and mouse, implied that different exons undergo different splicing frequency may be dependent on their instinct component features.

## Materials and Methods

### Database construction

Because about half of human genes, on average, up to four different transcript variants are produced by alternative splicing per gene and as a consequence translated into proteins of divergent biological functions [58]–[60]. To explore the regulatory mechanism for differently spliced exons, in the present work, we had selected alternatively spliced human genes with at least four isoforms according to the SWISS-PROT protein-sequence database (http://www.expasy.ch/sprot/). Transcripts and exonic sequences of each selected gene, together with orthologous mouse genes, were downloaded from the Ensembl database (version 46) (http://www.ensembl.org/). Based on their occurrence in all alternatively spliced transcriptional isoforms of relevant gene, exons were classified into four groups (G1∼G4) (also considering the above that the average number of transcript of nearly half of alternatively spliced human genes is four), with G1 including exons that only appear in one transcript of a gene, and G4 including exons that can be found in all transcripts of a gene (which also included the CSE group). Groups G2 (Should the number of isoforms of a gene be N, such exons can be found in N-2 transcripts of the gene with the splicing frequency = N-2) and G3 (Should the number of isoforms of a gene be N, such exons can be found in N-1 transcripts of the gene with the splicing frequency = N-1) are of the intermediates. A problem worthy to be pointed out is that we just wanted to investigate whether there are some tendencies in the splicing frequency of different exons, so we just classified exons into four categories based on the number of isoforms of the gene we selected. From the category of our studied exons we have shown that, for genes that have no more than four transcripts, we classified their exons into four categories based on their occurrence in the transcripts. For genes that have more than four transcripts, we have also classified their exons into four classes, especially with the aim to investigate the tendency of two extremes: G1 and G4 group. Moreover, our goal is to study the influences of ESE elements on splicing frequency. The probability for short exons contain ESE elements is too small, and there won't be distinct differences between these too short exons on the amounts of ESE elements, and it is also difficult to distinguish these short exons from each other in length. So short exons just influence the base number of exons and there maybe some noises in the ESE elements' influences of each exon group. Therefore, in order to eliminate noises, exons shorter than 11 nt were excluded. After filtration, 532 alternatively spliced human genes and 207 mouse orthologs were selected. For 126 mouse NAS genes, a total of 115 orthologous alternatively spliced human genes were retrieved (Table S3 and S4). Moreover, in order to investigate the relationships between exons' splicing frequency and genes' splicing pattern, we have selected five main splicing patterns (cassette exons, mutually exclusively exons, retained intron, alternative acceptor site and alternative donor site) of alternatively spliced human and orthologous mouse genes from the ASTRA database (http://alterna.cbrc.jp/index.php). Then, we selected our studied genes that corresponding to different exon groups with different splicing frequency from these original data for further investigate. We calculated the proportion value for each splicing pattern, and that is the number of genes corresponding to our studied each exon group to the number of our studied total human and mouse genes (Table S5), and then made statistical analysis.

### ESE extraction

The ESEfinder approach (version 3.0) (http://rulai.cshl.edu/tools/ESE/) [61] was adapted for extracting ESE elements from all selected exons. ESEfinder is a web resource for identifying putative exonic splicing enhancers responsive to the human SR proteins SF2/ASF, SC35, SRp40 and SRp55 using weight matrices. It also provides two types of putative splicing regulatory factors, one is based on SR proteins and the other is based on the splice site. Both are important for the recognition of splice site and regulation of alternative splicing. Based on spice site, ESEfinder provides five splicing regulatory factors, including BranchSite, 3′ splice sites (3′SS_U2_human, 3′SS_U2_mouse) and 5′ splice sites (5′SS_U2_human, 5′SS_U2_mouse). To simplify the analysis process, all the splicing regulatory factors (including putative exonic splicing enhancers and splicing site signals) were classified into six categories (named ESE_1 to ESE_6) base on their biological significance (Table S6).

### Repetitive elements

RepeatMasker (version Open 3-1-7) was used for detecting repetitive elements from human and mouse exonic sequences (http://www.repeatmasker.org/). The numbers of repetitive elements in each exonic group of alternatively spliced human genes and their mouse orthologs (Table 4), and in mouse NAS genes and alternatively spliced human orthologs were counted (Table S7).

### GC content

To further delineate the sequence characteristics of different exonic groups, GC content for different groups of human and mouse genes was calculated with a self-written Matlab program.

### Statistical analyses

The relationships between exons' splicing frequency and splicing pattern and various features of different exonic groups, including exon length, GC content, ESE element and repetitive element were analyzed using the SAS (statistical analysis system) program.

## Supporting Information

Table S1 Occurrence of ESE elements in different groups of human exons
(0.02 MB PDF)

Table S2 Occurrence of ESE elements in different groups of mouse exons
(0.02 MB PDF)

Table S3 Alternatively spliced human genes and mouse orthologs included in the study
(0.07 MB PDF)

Table S4 Alternatively spliced human genes and orthologous NAS mouse genes
(0.03 MB PDF)

Table S5 The proportions of each exon groups for each studied splicing pattern in alternatively spliced human and mouse genes
(0.02 MB PDF)

Table S6 Regulatory elements as divided into 6 main categories based on their biological functions
(0.02 MB PDF)

Table S7 Numbers of repetitive elements in different groups of NAS mouse exons and orthologous human exons
(0.02 MB PDF)
