# Supplementary material for: Comparative Component Analysis of Exons with Different Splicing Frequencies
Source: PLoS One. 2009 Apr 30;4(4):e5387. doi: 10.1371/journal.pone.0005387 (PMC2671145; doi:10.1371/journal.pone.0005387)
Supplement: Table S1 — Occurrence of ESE elements in different groups of human exons (0.02 MB PDF) [file pone.0005387.s001.pdf]

Table S1. Occurrence of ESE elements in different groups of human exons.

**exon group**	**G1**			**G2**			**G3**			**G4**		
	No.1	No.2	No.3	No.1	No.2	No.3	No.1	No.2	No.3	No.1	No.2	No.3
**ESE type**												
ESE_1	190360	5447	**34.95**	41405	1730	**23.93**	35932	1932	**18.6**	46610	3170	**14.7**
ESE_2	82776	5447	**15.2**	17665	1730	**10.21**	15525	1932	**8.04**	19915	3170	**6.28**
ESE_3	132477	5447	**24.32**	28561	1730	**16.51**	24531	1932	**12.7**	31889	3170	**10.06**
ESE_4	213298	5447	**39.16**	45386	1730	**26.23**	39078	1932	**20.23**	51275	3170	**16.17**
ESE_5	15324	5447	**2.81**	3062	1730	**1.77**	2424	1932	**1.25**	2640	3170	**0.83**
ESE_6	7760	5447	**1.42**	1394	1730	**0.805**	1070	1932	**0.55**	2356	3170	**0.74**

No.1: number of ESE in each exon group. No.2: number of exons in each different exon group. No.3: (values had been marked in bold): ESE frequencies in every exon group.
