# Supplementary material for: Comparative Component Analysis of Exons with Different Splicing Frequencies
Source: PLoS One. 2009 Apr 30;4(4):e5387. doi: 10.1371/journal.pone.0005387 (PMC2671145; doi:10.1371/journal.pone.0005387)
Supplement: Table S2 — Occurrence of ESE elements in different groups of mouse exons (0.02 MB PDF) [file pone.0005387.s002.pdf]

Table S2. Occurrence of ESE elements in different groups of mouse exons.

exon group	G1			G2			G3			G4		
	No.1	No.2	No.3	No.1	No.2	No.3	No.1	No.2	No.3	No.1	No.2	No.3
**ESE type**												
ESE_1	77615	1961	**39.58**	18849	826	**22.82**	28458	1359	**20.94**	21337	1307	**16.33**
ESE_2	34010	1961	**17.34**	7932	826	**9.6**	12396	1359	**9.12**	9143	1307	**7.00**
ESE_3	56228	1961	**28.67**	12551	826	**15.2**	20471	1359	**15.06**	14196	1307	**10.86**
ESE_4	89378	1961	**45.58**	19615	826	**23.75**	31534	1359	**23.2**	21871	1307	**16.73**
ESE_5	6493	1961	**3.31**	1183	826	**1.43**	1987	1359	**1.46**	1187	1307	**0.91**
ESE_6	2889	1961	**1.47**	644	826	**0.78**	878	1359	**0.65**	644	1307	**0.49**

No.1: number of ESE in each exon group. No.2: number of exons in each different exon group. No.3: (values had been marked in bold): ESE frequencies in every exon group.
