# Supplementary material for: Comparative Component Analysis of Exons with Different Splicing Frequencies
Source: PLoS One. 2009 Apr 30;4(4):e5387. doi: 10.1371/journal.pone.0005387 (PMC2671145; doi:10.1371/journal.pone.0005387)
Supplement: Table S3 — Alternatively spliced human genes and mouse orthologs included in the study (0.07 MB PDF) [file pone.0005387.s003.pdf]

Table S3. Alternatively spliced human genes and mouse orthologs included in the study.

Species			
Alternatively spliced human genes			Alternatively spliced mouse genes
**SwissProt Name**	**SwissProt(acc)**	**Human Ensembl_Gene ID**	**Mouse Ensembl_Gene ID**
LAS1L_HUMAN	Q9Y4W2	ENSG00000001497	
CFLAR_HUMAN	O15519	ENSG00000003402	ENSMUSG00000026031
TCGAP_HUMAN	O14559	ENSG00000004777	
CAC1G_HUMAN	O43497	ENSG00000006283	ENSMUSG00000020866
CD2L2_HUMAN	Q9UQ88	ENSG00000008128	ENSMUSG00000029062
CD2L1_HUMAN	P21127	ENSG00000008128	
JIP4_HUMAN	O60271	ENSG00000008294	ENSMUSG00000020859
NFIX_HUMAN	Q14938	ENSG00000008441	ENSMUSG00000001911
FA76A_HUMAN	Q8TAV0	ENSG00000009780	
ETV7_HUMAN	Q9Y603	ENSG00000010030	
K0859_HUMAN	Q8N6R0	ENSG00000010165	
HFE_HUMAN	Q30201	ENSG00000010704	ENSMUSG00000006611
SCMH1_HUMAN	Q96GD3	ENSG00000010803	ENSMUSG00000000085
CLCN6_HUMAN	P51797	ENSG00000011021	
PGS2_HUMAN	P07585	ENSG00000011465	
DDX11_HUMAN	Q96FC9	ENSG00000013573	
CD6_HUMAN	P30203	ENSG00000013725	ENSMUSG00000024670
BID_HUMAN	P55957	ENSG00000015475	
ZF64A_HUMAN	Q9NPA5	ENSG00000020256	ENSMUSG00000027551
ZF64B_HUMAN	Q9NTW7	ENSG00000020256	
CP343_HUMAN	Q9HB55	ENSG00000021461	
UBR2_HUMAN	Q8IWV8	ENSG00000024048	
RTEL1_HUMAN	Q9NZ71	ENSG00000026036	ENSMUSG00000038685
TNR6_HUMAN	P25445	ENSG00000026103	
PO2F2_HUMAN	P09086	ENSG00000028277	ENSMUSG00000008496
BRD9_HUMAN	Q9H8M2	ENSG00000028310	
ANK1_HUMAN	P16157	ENSG00000029534	ENSMUSG00000031543
S4A7_HUMAN	Q9Y6M7	ENSG00000033867	ENSMUSG00000021733
CSPG2_HUMAN	P13611	ENSG00000038427	ENSMUSG00000021614
TENA_HUMAN	P24821	ENSG00000041982	ENSMUSG00000028364
MOD5_HUMAN	Q9H3H1	ENSG00000043514	
RHG06_HUMAN	O43182	ENSG00000047648	ENSMUSG00000031355
F120A_HUMAN	Q9NZB2	ENSG00000048828	
ELN_HUMAN	P15502	ENSG00000049540	
NED4L_HUMAN	Q96PU5	ENSG00000049759	ENSMUSG00000024589

PE2R3_HUMAN	P43115	ENSG00000050628	
PKHA5_HUMAN	Q9HAU0	ENSG00000052126	
HHAT_HUMAN	Q5VTY9	ENSG00000054392	ENSMUSG00000037375
KIF1B_HUMAN	O60333	ENSG00000054523	ENSMUSG00000063077
SYNE2_HUMAN	Q8WXH0	ENSG00000054654	
CRDL2_HUMAN	Q6WN34	ENSG00000054938	
RM43_HUMAN	Q8N983	ENSG00000055950	
MNAB_HUMAN	Q9HBD1	ENSG00000056586	
GLYG2_HUMAN	O15488	ENSG00000056998	
MYPT1_HUMAN	O14974	ENSG00000058272	
KCC2B_HUMAN	Q13554	ENSG00000058404	ENSMUSG00000057897
AT2B4_HUMAN	P23634	ENSG00000058668	ENSMUSG00000026463
HDAC7_HUMAN	Q8WUI4	ENSG00000061273	
LZTS1_HUMAN	Q9Y250	ENSG00000061337	
CASP8_HUMAN	Q14790	ENSG00000064012	
TSN32_HUMAN	Q96QS1	ENSG00000064201	ENSMUSG00000000244
AP3D1_HUMAN	O14617	ENSG00000065000	
MYLK_HUMAN	Q15746	ENSG00000065534	ENSMUSG00000022836
PDE4A_HUMAN	P27815	ENSG00000065989	ENSMUSG00000032177
FGFR2_HUMAN	P21802	ENSG00000066468	ENSMUSG00000030849
AT2B3_HUMAN	Q16720	ENSG00000067842	ENSMUSG00000031376
RASF1_HUMAN	Q9NS23	ENSG00000068028	
GRAP1_HUMAN	Q4V328	ENSG00000068400	ENSMUSG00000031153
M4K4_HUMAN	O95819	ENSG00000071054	
MYO3B_HUMAN	Q8WXR4	ENSG00000071909	ENSMUSG00000042064
ACCN4_HUMAN	Q96FT7	ENSG00000072182	
AFF4_HUMAN	Q9UHB7	ENSG00000072364	
MARK2_HUMAN	Q7KZI7	ENSG00000072518	ENSMUSG00000024969
NFAC3_HUMAN	Q12968	ENSG00000072736	
SCRB1_HUMAN	Q8WTV0	ENSG00000073060	
P73L_HUMAN	Q9H3D4	ENSG00000073282	ENSMUSG00000022510
GLI2_HUMAN	P10070	ENSG00000074047	
DPP8_HUMAN	Q6V1X1	ENSG00000074603	
KCNQ2_HUMAN	O43526	ENSG00000075043	ENSMUSG00000016346
ADDB_HUMAN	P35612	ENSG00000075340	
MARK3_HUMAN	P27448	ENSG00000075413	
PLD1_HUMAN	Q13393	ENSG00000075651	
DLG1_HUMAN	Q12959	ENSG00000075711	ENSMUSG00000022770
MBN3_HUMAN	Q9NUK0	ENSG00000076770	
MYPT2_HUMAN	O60237	ENSG00000077157	ENSMUSG00000073557
DCX_HUMAN	O43602	ENSG00000077279	ENSMUSG00000031285
SPAG6_HUMAN	O75602	ENSG00000077327	

SIRT6_HUMAN	Q8N6T7	ENSG00000077463	
FGFR1_HUMAN	P11362	ENSG00000077782	
FBLN1_HUMAN	P23142	ENSG00000077942	
ZCPW1_HUMAN	Q9H0M4	ENSG00000078487	
MTG8R_HUMAN	O43439	ENSG00000078699	ENSMUSG00000038533
PK2L2_HUMAN	Q9NZM6	ENSG00000078795	
P73_HUMAN	O15350	ENSG00000078900	ENSMUSG00000029026
CC14A_HUMAN	Q9UNH5	ENSG00000079335	
CEAM1_HUMAN	P13688	ENSG00000079385	
RIMS1_HUMAN	Q86UR5	ENSG00000079841	ENSMUSG00000041670
CNOT4_HUMAN	O95628	ENSG00000080802	
PSN1_HUMAN	P49768	ENSG00000080815	
TCF7_HUMAN	P36402	ENSG00000081059	
PCDGK_HUMAN	Q9UN70	ENSG00000081853	ENSMUSG00000023036
MPP4_HUMAN	Q96JB8	ENSG00000082126	ENSMUSG00000079550
RB6I2_HUMAN	Q8IUD2	ENSG00000082805	ENSMUSG00000030172
TRPM3_HUMAN	Q9HCF6	ENSG00000083067	ENSMUSG00000052387
ZCHC6_HUMAN	Q5VYS8	ENSG00000083223	
SSH1_HUMAN	Q8WYL5	ENSG00000084112	
ATRX_HUMAN	P46100	ENSG00000085224	ENSMUSG00000031229
A16L1_HUMAN	Q676U5	ENSG00000085978	ENSMUSG00000026289
FOLH1_HUMAN	Q04609	ENSG00000086205	
PPE1_HUMAN	O14829	ENSG00000086717	
BAXA_HUMAN	Q07812	ENSG00000087088	
BAXB_HUMAN	Q07814	ENSG00000087088	
BAXC_HUMAN	Q07815	ENSG00000087088	
BAXD_HUMAN	P55269	ENSG00000087088	
RXINP_HUMAN	Q96RL1	ENSG00000087206	
DNM1L_HUMAN	O00429	ENSG00000087470	ENSMUSG00000022789
DNM3B_HUMAN	Q9UBC3	ENSG00000088305	ENSMUSG00000027478
DOCK9_HUMAN	Q9BZ29	ENSG00000088387	
SMOX_HUMAN	Q9NWM0	ENSG00000088826	ENSMUSG00000027333
CT012_HUMAN	Q9NVP4	ENSG00000089091	
CT026_HUMAN	Q8NHU2	ENSG00000089101	
SNX23_HUMAN	Q96L93	ENSG00000089177	
CKLF1_HUMAN	Q8IZ96	ENSG00000089505	ENSMUSG00000054400
CHRD_HUMAN	Q9H2X0	ENSG00000090539	
CD209_HUMAN	Q9NNX6	ENSG00000090659	
NRCAM_HUMAN	Q92823	ENSG00000091129	ENSMUSG00000020598
ITA6_HUMAN	P23229	ENSG00000091409	
NALP1_HUMAN	Q9C000	ENSG00000091592	
NALP1_HUMAN	Q9C000	ENSG00000091592	ENSMUSG00000070390

NALP1_HUMAN	Q9C000	ENSG00000091592	
CAN3_HUMAN	P20807	ENSG00000092529	ENSMUSG00000079110
SH2D3_HUMAN	Q8N5H7	ENSG00000095370	
WHRN_HUMAN	Q9P202	ENSG00000095397	ENSMUSG00000039137
SRBS1_HUMAN	Q9BX66	ENSG00000095637	
CREM_HUMAN	Q03060	ENSG00000095794	ENSMUSG00000063889
BACH_HUMAN	O00154	ENSG00000097021	
ABLM1_HUMAN	O14639	ENSG00000099204	ENSMUSG00000025085
PC11Y_HUMAN	Q9BZA8	ENSG00000099715	ENSMUSG00000034755
PATZ1_HUMAN	Q9HBE1	ENSG00000100105	ENSMUSG00000020453
DEPD5_HUMAN	O75140	ENSG00000100150	ENSMUSG00000037426
MTMR5_HUMAN	O95248	ENSG00000100241	
RBM23_HUMAN	Q86U06	ENSG00000100461	
NIN_HUMAN	Q8N4C6	ENSG00000100503	
BZFB_HUMAN	Q9NVA1	ENSG00000101019	ENSMUSG00000005882
PKCB1_HUMAN	Q9ULU4	ENSG00000101040	ENSMUSG00000039671
HNF4A_HUMAN	P41235	ENSG00000101076	
SLAP2_HUMAN	Q9H6Q3	ENSG00000101082	
HRH3_HUMAN	Q9Y5N1	ENSG00000101180	
SO4A1_HUMAN	Q96BD0	ENSG00000101187	
DIDO1_HUMAN	Q9BTC0	ENSG00000101191	ENSMUSG00000038914
MPIP2_HUMAN	P30305	ENSG00000101224	
CDS2_HUMAN	O95674	ENSG00000101290	
HM13_HUMAN	Q8TCT9	ENSG00000101294	ENSMUSG00000019188
CK5P1_HUMAN	Q96SZ6	ENSG00000101391	
WFDC2_HUMAN	Q14508	ENSG00000101443	ENSMUSG00000017723
WFDC2_HUMAN	Q14508	ENSG00000101443	
WFDC2_HUMAN	Q14508	ENSG00000101443	
AT11C_HUMAN	Q8NB49	ENSG00000101974	
MCF2_HUMAN	P10911	ENSG00000101977	
FMR1_HUMAN	Q06787	ENSG00000102081	ENSMUSG00000000838
PQBP1_HUMAN	O60828	ENSG00000102103	ENSMUSG00000031157
TAZ_HUMAN	Q16635	ENSG00000102125	ENSMUSG00000009995
PC11X_HUMAN	Q9BZA7	ENSG00000102290	
PORCN_HUMAN	Q9H237	ENSG00000102312	ENSMUSG00000031169
CM018_HUMAN	Q9H714	ENSG00000102445	
ARHG7_HUMAN	Q14155	ENSG00000102606	ENSMUSG00000031511
DGKH_HUMAN	Q86XP1	ENSG00000102780	
NDRG4_HUMAN	Q9ULP0	ENSG00000103034	
TSC2_HUMAN	P49815	ENSG00000103197	ENSMUSG00000002496
MRP1_HUMAN	P33527	ENSG00000103222	
ARHGA_HUMAN	O15013	ENSG00000104728	

DMPK_HUMAN	Q09013	ENSG00000104936	ENSMUSG00000030409
CLC4M_HUMAN	Q9H2X3	ENSG00000104938	
HNRL1_HUMAN	Q9BUJ2	ENSG00000105323	
MYH14_HUMAN	Q7Z406	ENSG00000105357	
PTPRS_HUMAN	Q13332	ENSG00000105426	ENSMUSG00000013236
HPS1_HUMAN	Q92902	ENSG00000107521	
RASF4_HUMAN	Q9H2L5	ENSG00000107551	
PK2L1_HUMAN	Q9P0L9	ENSG00000107593	
TRDMT_HUMAN	O14717	ENSG00000107614	
MK08_HUMAN	P45983	ENSG00000107643	ENSMUSG00000021936
FGF8_HUMAN	P55075	ENSG00000107831	ENSMUSG00000025219
CMTA2_HUMAN	O94983	ENSG00000108509	ENSMUSG00000040712
RAI1_HUMAN	Q7Z5J4	ENSG00000108557	ENSMUSG00000062115
ELF2_HUMAN	Q15723	ENSG00000109381	ENSMUSG00000037174
KKCC2_HUMAN	Q96RR4	ENSG00000110931	ENSMUSG00000029471
SCNNA_HUMAN	P37088	ENSG00000111319	
CLC4A_HUMAN	Q9UMR7	ENSG00000111729	
NCOA7_HUMAN	Q8NI08	ENSG00000111912	ENSMUSG00000039697
EYA4_HUMAN	O95677	ENSG00000112319	
SNX3_HUMAN	O60493	ENSG00000112335	
QKI_HUMAN	Q96PU8	ENSG00000112531	ENSMUSG00000062078
VEGFA_HUMAN	P15692	ENSG00000112715	ENSMUSG00000023951
LAP2_HUMAN	Q96RT1	ENSG00000112851	
APBB3_HUMAN	O95704	ENSG00000113108	
PDE8B_HUMAN	O95263	ENSG00000113231	
PDE4D_HUMAN	Q08499	ENSG00000113448	
NPHP3_HUMAN	Q7Z494	ENSG00000113971	ENSMUSG00000032558
OGG1_HUMAN	O15527	ENSG00000114026	
FOXP1_HUMAN	Q9H334	ENSG00000114861	ENSMUSG00000030067
IF4G1_HUMAN	Q04637	ENSG00000114867	ENSMUSG00000045983
DGUOK_HUMAN	Q16854	ENSG00000114956	
OTOF_HUMAN	Q9HC10	ENSG00000115155	
PDE1A_HUMAN	P54750	ENSG00000115252	ENSMUSG00000059173
RTN4_HUMAN	Q9NQC3	ENSG00000115310	ENSMUSG00000020458
FINC_HUMAN	P02751	ENSG00000115414	
PERT_HUMAN	P07202	ENSG00000115705	
PAR3L_HUMAN	Q8TEW8	ENSG00000116117	ENSMUSG00000052062
AMPD2_HUMAN	Q01433	ENSG00000116337	ENSMUSG00000027889
MEF2D_HUMAN	Q14814	ENSG00000116604	
LEPR_HUMAN	P48357	ENSG00000116678	ENSMUSG00000057722
PRG4_HUMAN	Q92954	ENSG00000116690	ENSMUSG00000006014
SMG7_HUMAN	Q92540	ENSG00000116698	

UCHL5_HUMAN	Q9Y5K5	ENSG00000116750	
TNI3K_HUMAN	Q59H18	ENSG00000116783	
LPHN2_HUMAN	O95490	ENSG00000117114	ENSMUSG00000028184
ECE1_HUMAN	P42892	ENSG00000117298	
CR2_HUMAN	P20023	ENSG00000117322	
MCP_HUMAN	P15529	ENSG00000117335	
DPH5_HUMAN	Q9H2P9	ENSG00000117543	
PTBP2_HUMAN	Q9UKA9	ENSG00000117569	
TNNT2_HUMAN	P45379	ENSG00000118194	ENSMUSG00000026414
SGIP1_HUMAN	Q9BQI5	ENSG00000118473	ENSMUSG00000028524
MYB_HUMAN	P10242	ENSG00000118513	
TRPM6_HUMAN	Q9BX84	ENSG00000119121	
ZO2_HUMAN	Q9UDY2	ENSG00000119139	
PTPA_HUMAN	Q15257	ENSG00000119383	
CSF3R_HUMAN	Q99062	ENSG00000119535	
BC11A_HUMAN	Q9H165	ENSG00000119866	ENSMUSG00000000861
TECT3_HUMAN	Q6NUS6	ENSG00000119977	
KCIP2_HUMAN	Q9NS61	ENSG00000120049	ENSMUSG00000025221
CRFR1_HUMAN	P34998	ENSG00000120088	
EPC1_HUMAN	Q9H2F5	ENSG00000120616	ENSMUSG00000024240
APAF_HUMAN	O14727	ENSG00000120868	
ADA1A_HUMAN	P35348	ENSG00000120907	
PDLI2_HUMAN	Q96JY6	ENSG00000120913	
POLK_HUMAN	Q9UBT6	ENSG00000122008	ENSMUSG00000021668
OBP2A_HUMAN	Q9NY56	ENSG00000122136	
OBP2A_HUMAN	Q9NY56	ENSG00000122136	
OBP2A_HUMAN	Q9NY56	ENSG00000122136	
OBP2A_HUMAN	Q9NY56	ENSG00000122136	
CD244_HUMAN	Q9BZW8	ENSG00000122223	
RBBP6_HUMAN	Q7Z6E9	ENSG00000122257	ENSMUSG00000030779
LDB3_HUMAN	O75112	ENSG00000122367	ENSMUSG00000021798
PTHB1_HUMAN	Q3SYG4	ENSG00000122507	
NCOA3_HUMAN	Q9Y6Q9	ENSG00000124151	
PIGT_HUMAN	Q969N2	ENSG00000124155	
MATN4_HUMAN	O95460	ENSG00000124159	ENSMUSG00000017721
UB2V1_HUMAN	Q13404	ENSG00000124208	ENSMUSG00000016995
CAD26_HUMAN	Q8IXH8	ENSG00000124215	
STX16_HUMAN	O14662	ENSG00000124222	
ZBP1_HUMAN	Q9H171	ENSG00000124256	
WRIP1_HUMAN	Q96S55	ENSG00000124535	
MOS1A_HUMAN	O14940	ENSG00000124615	
MOS1B_HUMAN	Q9NZB8	ENSG00000124615	

SEPT6_HUMAN	Q14141	ENSG00000125354	ENSMUSG00000050379
CD008_HUMAN	P78312	ENSG00000125386	
DDX31_HUMAN	Q9H8H2	ENSG00000125485	
IL1F7_HUMAN	Q9NZH6	ENSG00000125571	
PAX8_HUMAN	Q06710	ENSG00000125618	
UB7I3_HUMAN	Q9BYM8	ENSG00000125826	
SIAT6_HUMAN	Q11203	ENSG00000126091	ENSMUSG00000028538
KLC1_HUMAN	Q07866	ENSG00000126214	ENSMUSG00000021288
PCID2_HUMAN	Q5JVF3	ENSG00000126226	
KIRR2_HUMAN	Q6UWL6	ENSG00000126259	
MIRO1_HUMAN	Q8IXI2	ENSG00000126858	ENSMUSG00000017686
IBA2_HUMAN	Q9BQI0	ENSG00000126878	
NXF5_HUMAN	Q9H1B4	ENSG00000126952	
BEST3_HUMAN	Q8N1M1	ENSG00000127325	
RAB3I_HUMAN	Q96QF0	ENSG00000127328	
K0090_HUMAN	Q8N766	ENSG00000127463	
MACF1_HUMAN	Q9UPN3	ENSG00000127603	ENSMUSG00000028649
MACF4_HUMAN	Q96PK2	ENSG00000127603	
AKAP9_HUMAN	Q99996	ENSG00000127914	
STYL1_HUMAN	Q9Y6J8	ENSG00000127952	ENSMUSG00000019178
FOXP2_HUMAN	O15409	ENSG00000128573	ENSMUSG00000029563
PDE11_HUMAN	Q9HCR9	ENSG00000128655	ENSMUSG00000075270
ILF3_HUMAN	Q12906	ENSG00000129351	
AFAD_HUMAN	P55196	ENSG00000130396	
NOE1_HUMAN	Q99784	ENSG00000130558	ENSMUSG00000026833
TNNT3_HUMAN	P45378	ENSG00000130595	ENSMUSG00000061723
ICB1_HUMAN	Q5TEJ8	ENSG00000130775	
SYNE1_HUMAN	Q8NF91	ENSG00000131018	ENSMUSG00000019769
PSG1_HUMAN	P11464	ENSG00000131113	
FCRLA_HUMAN	Q7L513	ENSG00000132185	
TRIM5_HUMAN	Q9C035	ENSG00000132256	
TRIM5_HUMAN	Q9C035	ENSG00000132256	ENSMUSG00000060441
TRIM5_HUMAN	Q9C035	ENSG00000132256	
TRIM5_HUMAN	Q9C035	ENSG00000132256	
TRIM5_HUMAN	Q9C035	ENSG00000132256	
TRIM5_HUMAN	Q9C035	ENSG00000132256	
TRIM5_HUMAN	Q9C035	ENSG00000132256	ENSMUSG00000066258
F113A_HUMAN	Q9H1Q7	ENSG00000132635	
PTPRA_HUMAN	P18433	ENSG00000132670	
FCRL2_HUMAN	Q96LA5	ENSG00000132704	
MUTYH_HUMAN	Q9UIF7	ENSG00000132781	
INADL_HUMAN	Q8NI35	ENSG00000132849	ENSMUSG00000061859

TPTE2_HUMAN	Q6XPS3	ENSG00000132958	
DCAK1_HUMAN	O15075	ENSG00000133083	ENSMUSG00000027797
TRPC4_HUMAN	Q9UBN4	ENSG00000133107	
STA13_HUMAN	Q9Y3M8	ENSG00000133121	
BMAL1_HUMAN	O00327	ENSG00000133794	
CN159_HUMAN	Q7Z3D6	ENSG00000133943	ENSMUSG00000021185
NUMB_HUMAN	P49757	ENSG00000133961	ENSMUSG00000021224
MEIS2_HUMAN	O14770	ENSG00000134138	ENSMUSG00000027210
PPHLN_HUMAN	Q8NEY8	ENSG00000134283	
PPHLN_HUMAN	Q8NEY8	ENSG00000134283	
PPHLN_HUMAN	Q8NEY8	ENSG00000134283	
CRUM1_HUMAN	P82279	ENSG00000134376	ENSMUSG00000063681
I15RA_HUMAN	Q13261	ENSG00000134470	ENSMUSG00000023206
DDB2_HUMAN	Q92466	ENSG00000134574	
DTNA_HUMAN	Q9Y4J8	ENSG00000134769	ENSMUSG00000024302
ASPX_HUMAN	P26436	ENSG00000134940	
MTO1_HUMAN	Q9Y2Z2	ENSG00000135297	
ITA7_HUMAN	Q13683	ENSG00000135424	ENSMUSG00000025348
MDM2_HUMAN	Q00987	ENSG00000135679	
SP110_HUMAN	Q9HB58	ENSG00000135899	
SP110_HUMAN	Q9HB58	ENSG00000135899	
FLNB_HUMAN	O75369	ENSG00000136068	
LMO7_HUMAN	Q8WWI1	ENSG00000136153	
RASF5_HUMAN	Q8WWW0	ENSG00000136653	ENSMUSG00000026430
BIN1_HUMAN	O00499	ENSG00000136717	
ABI1_HUMAN	Q8IZP0	ENSG00000136754	ENSMUSG00000058835
CK5P2_HUMAN	Q96SN8	ENSG00000136861	
APTX_HUMAN	Q7Z2E3	ENSG00000137074	ENSMUSG00000028411
DCTN3_HUMAN	O75935	ENSG00000137100	
AP2A_HUMAN	P05549	ENSG00000137203	ENSMUSG00000021359
TJAP1_HUMAN	Q5JTD0	ENSG00000137221	
MYO7A_HUMAN	Q13402	ENSG00000137474	ENSMUSG00000030761
SYTL2_HUMAN	Q9HCH5	ENSG00000137501	ENSMUSG00000030616
CASP1_HUMAN	P29466	ENSG00000137752	
SEM6D_HUMAN	Q8NFY4	ENSG00000137872	ENSMUSG00000027200
DTNB_HUMAN	O60941	ENSG00000138101	
CUZD1_HUMAN	Q86UP6	ENSG00000138161	
TALAN_HUMAN	Q70YC4	ENSG00000138311	
ZN365_HUMAN	Q70YC5	ENSG00000138311	
HNRPD_HUMAN	Q14103	ENSG00000138668	ENSMUSG00000000568
FRAS1_HUMAN	Q86XX4	ENSG00000138759	
IPYR2_HUMAN	Q9H2U2	ENSG00000138777	

RGS3_HUMAN	P49796	ENSG00000138835	ENSMUSG00000059810
RGS3_HUMAN	P49796	ENSG00000138835	
GIT2_HUMAN	Q14161	ENSG00000139436	ENSMUSG00000041890
ESR2_HUMAN	Q92731	ENSG00000140009	ENSMUSG00000021055
TPM1_HUMAN	P09493	ENSG00000140416	ENSMUSG00000032366
PML_HUMAN	P29590	ENSG00000140464	
PCSK6_HUMAN	P29122	ENSG00000140479	
NTRK3_HUMAN	Q16288	ENSG00000140538	
BCAS3_HUMAN	Q9H6U6	ENSG00000141376	ENSMUSG00000059439
TBCD_HUMAN	Q9BTW9	ENSG00000141556	
MBD1_HUMAN	Q9UIS9	ENSG00000141644	
CAC1A_HUMAN	O00555	ENSG00000141837	
NFIC_HUMAN	P08651	ENSG00000141905	ENSMUSG00000055053
DPP9_HUMAN	Q86TI2	ENSG00000142002	
A4_HUMAN	P05067	ENSG00000142192	
CAN10_HUMAN	Q9HC96	ENSG00000142330	
NAL12_HUMAN	P59046	ENSG00000142405	
PAIRB_HUMAN	Q8NC51	ENSG00000142864	
KAPCB_HUMAN	P22694	ENSG00000142875	ENSMUSG00000005034
ADC_HUMAN	Q96A70	ENSG00000142920	ENSMUSG00000028789
PO2F1_HUMAN	P14859	ENSG00000143190	ENSMUSG00000026565
FCRL5_HUMAN	Q96RD9	ENSG00000143297	
POGZ_HUMAN	Q7Z3K3	ENSG00000143442	ENSMUSG00000038902
SYT14_HUMAN	Q8NB59	ENSG00000143469	
VASH2_HUMAN	Q86V25	ENSG00000143494	
CA043_HUMAN	Q9BWL3	ENSG00000143612	
MRCKA_HUMAN	Q5VT25	ENSG00000143776	ENSMUSG00000026490
HNRLL_HUMAN	Q8WVV9	ENSG00000143889	
SPG16_HUMAN	Q8N0X2	ENSG00000144451	ENSMUSG00000053153
GOGA4_HUMAN	Q13439	ENSG00000144674	
KCC2D_HUMAN	Q13557	ENSG00000145349	ENSMUSG00000053819
AMD_HUMAN	P19021	ENSG00000145730	
AIG1_HUMAN	Q9NVV5	ENSG00000146416	
TACC1_HUMAN	O75410	ENSG00000147526	
TRI55_HUMAN	Q9BYV6	ENSG00000147573	
CD2A1_HUMAN	P42771	ENSG00000147889	
CD2A2_HUMAN	Q8N726	ENSG00000147889	
CBWD1_HUMAN	Q9BRT8	ENSG00000147996	
AMPO_HUMAN	Q8N6M6	ENSG00000148120	
RRFM_HUMAN	Q96E11	ENSG00000148187	
PARD3_HUMAN	Q8TEW0	ENSG00000148498	
ACF_HUMAN	Q9NQ94	ENSG00000148584	

KCC2G_HUMAN	Q13555	ENSG00000148660	ENSMUSG00000021820
F10C1_HUMAN	Q70Z53	ENSG00000148690	
TF7L2_HUMAN	Q9NQB0	ENSG00000148737	ENSMUSG00000024985
LRC27_HUMAN	Q9C0I9	ENSG00000148814	
PAOX_HUMAN	Q6QHF9	ENSG00000148832	
TMM25_HUMAN	Q86YD3	ENSG00000149582	
KLRF1_HUMAN	Q9NZS2	ENSG00000150045	
ITB1_HUMAN	P05556	ENSG00000150093	
CTGE5_HUMAN	O15320	ENSG00000150527	
ITPR1_HUMAN	Q14643	ENSG00000150995	ENSMUSG00000030102
MAGI1_HUMAN	Q96QZ7	ENSG00000151276	ENSMUSG00000045095
NPAS3_HUMAN	Q8IXF0	ENSG00000151322	
MCR_HUMAN	P08235	ENSG00000151623	ENSMUSG00000031618
BPA1_HUMAN	Q03001	ENSG00000151914	ENSMUSG00000026131
BPAEA_HUMAN	O94833	ENSG00000151914	
BPAEB_HUMAN	Q8WXK8	ENSG00000151914	
DCR1C_HUMAN	Q96SD1	ENSG00000152457	ENSMUSG00000026648
PANK1_HUMAN	Q8TE04	ENSG00000152782	
RAD17_HUMAN	O75943	ENSG00000152942	
ICAL_HUMAN	P20810	ENSG00000153113	ENSMUSG00000021585
SCN3A_HUMAN	Q9NY46	ENSG00000153253	ENSMUSG00000057182
ING1_HUMAN	Q9UK53	ENSG00000153487	
TARSH_HUMAN	Q7Z7G0	ENSG00000154175	ENSMUSG00000035258
LRRK1_HUMAN	Q38SD2	ENSG00000154237	ENSMUSG00000015133
TNIK_HUMAN	Q9UKE5	ENSG00000154310	
OBSCN_HUMAN	Q5VST9	ENSG00000154358	ENSMUSG00000061462
CXAR_HUMAN	P78310	ENSG00000154639	
FXL18_HUMAN	Q96ME1	ENSG00000155034	
MMS19_HUMAN	Q96T76	ENSG00000155229	
ZFY27_HUMAN	Q5T4F4	ENSG00000155256	
TITIN_HUMAN	Q8WZ42	ENSG00000155657	
AFF2_HUMAN	P51816	ENSG00000155966	
KCMA1_HUMAN	Q12791	ENSG00000156113	ENSMUSG00000063142
RPGR_HUMAN	Q92834	ENSG00000156313	ENSMUSG00000031174
HXK1_HUMAN	P19367	ENSG00000156515	
AT2B2_HUMAN	Q01814	ENSG00000157087	ENSMUSG00000030302
SMG1_HUMAN	Q96Q15	ENSG00000157106	
RBPMS_HUMAN	Q93062	ENSG00000157110	
NRG1_HUMAN	Q02297	ENSG00000157168	
SMDF_HUMAN	Q15491	ENSG00000157168	
LRP8_HUMAN	Q14114	ENSG00000157193	ENSMUSG00000028613
GRHL3_HUMAN	Q8TE85	ENSG00000158055	

S13A3_HUMAN	Q8WWT9	ENSG00000158296	
MPIP3_HUMAN	P30307	ENSG00000158402	
NRG2_HUMAN	O14511	ENSG00000158458	ENSMUSG00000060275
KAT_HUMAN	Q8NFU3	ENSG00000158769	
EDA_HUMAN	Q92838	ENSG00000158813	ENSMUSG00000059327
41_HUMAN	P11171	ENSG00000159023	ENSMUSG00000028906
GCFC_HUMAN	Q9Y5B6	ENSG00000159086	
SON_HUMAN	P18583	ENSG00000159140	
RUNX1_HUMAN	Q01196	ENSG00000159216	ENSMUSG00000022952
RGS12_HUMAN	O14924	ENSG00000159788	ENSMUSG00000029101
ABCG1_HUMAN	P45844	ENSG00000160179	
PDE9A_HUMAN	O76083	ENSG00000160191	
TOR2A_HUMAN	Q5JU69	ENSG00000160404	
TOR2X_HUMAN	Q8N2E6	ENSG00000160404	
PAQR6_HUMAN	Q6TCH4	ENSG00000160781	
PMF1_HUMAN	Q6P1K2	ENSG00000160783	
CASP_HUMAN	Q13948	ENSG00000160967	
CUTL1_HUMAN	P39880	ENSG00000160967	
FXL13_HUMAN	Q8NEE6	ENSG00000161040	
AIOL_HUMAN	Q9UKT9	ENSG00000161405	ENSMUSG00000059857
NTNG1_HUMAN	Q9Y2I2	ENSG00000162631	ENSMUSG00000032691
CIAS1_HUMAN	Q96P20	ENSG00000162711	
DISC1_HUMAN	Q9NRI5	ENSG00000162946	
PAQR3_HUMAN	Q6TCH7	ENSG00000163291	
HIPK1_HUMAN	Q86Z02	ENSG00000163349	
YYAP1_HUMAN	Q9H869	ENSG00000163374	
TRI46_HUMAN	Q7Z4K8	ENSG00000163462	ENSMUSG00000042766
NFASC_HUMAN	O94856	ENSG00000163531	
CAPS1_HUMAN	Q9ULU8	ENSG00000163618	
I17RC_HUMAN	Q8NAC3	ENSG00000163702	
PB1_HUMAN	Q86U86	ENSG00000163939	ENSMUSG00000042323
ABLM2_HUMAN	Q6H8Q1	ENSG00000163995	ENSMUSG00000029095
ATRIP_HUMAN	Q8WXE1	ENSG00000164053	
5HT4R_HUMAN	Q13639	ENSG00000164270	
GLT10_HUMAN	Q86SR1	ENSG00000164574	
KIF6_HUMAN	Q6ZMV9	ENSG00000164627	
TAGAP_HUMAN	Q8N103	ENSG00000164691	
TAGAP_HUMAN	Q8N103	ENSG00000164691	
TAGAP_HUMAN	Q8N103	ENSG00000164691	
CSMD3_HUMAN	Q7Z407	ENSG00000164796	ENSMUSG00000022311
UN84A_HUMAN	O94901	ENSG00000164828	ENSMUSG00000036817
SPG11_HUMAN	Q08648	ENSG00000164871	

BAALC_HUMAN	Q8WXS3	ENSG00000164929	
FREM1_HUMAN	Q5H8C1	ENSG00000164946	
ANKS6_HUMAN	Q68DC2	ENSG00000165138	
WNK2_HUMAN	Q9Y3S1	ENSG00000165238	ENSMUSG00000037989
ATP7A_HUMAN	Q04656	ENSG00000165240	
NOL6_HUMAN	Q9H6R4	ENSG00000165271	
TTC8_HUMAN	Q8TAM2	ENSG00000165533	
VDAC2_HUMAN	P45880	ENSG00000165637	
DACH1_HUMAN	Q9UI36	ENSG00000165659	ENSMUSG00000055639
NDRG2_HUMAN	Q9UN36	ENSG00000165795	
NELF_HUMAN	Q6X4W1	ENSG00000165802	ENSMUSG00000006476
CACB2_HUMAN	Q08289	ENSG00000165995	ENSMUSG00000057914
CKLF5_HUMAN	Q96DZ9	ENSG00000166091	
TPTE_HUMAN	P56180	ENSG00000166157	
NKX31_HUMAN	Q99801	ENSG00000167034	
PBX3_HUMAN	P40426	ENSG00000167081	ENSMUSG00000038718
NALDL_HUMAN	Q9UQQ1	ENSG00000168060	
SF01_HUMAN	Q15637	ENSG00000168066	ENSMUSG00000024949
PXK_HUMAN	Q7Z7A4	ENSG00000168297	
BMP1_HUMAN	P13497	ENSG00000168487	ENSMUSG00000022098
ATX2L_HUMAN	Q8WWM7	ENSG00000168488	
DFFB_HUMAN	O76075	ENSG00000169598	
CNTRB_HUMAN	Q8N137	ENSG00000170037	
S26A5_HUMAN	P58743	ENSG00000170615	
ZN692_HUMAN	Q9BU19	ENSG00000171163	
ZDH16_HUMAN	Q969W1	ENSG00000171307	
TNR25_HUMAN	Q93038	ENSG00000171680	ENSMUSG00000039713
MAL_HUMAN	P21145	ENSG00000172005	
CD8B_HUMAN	P10966	ENSG00000172116	
NARGL_HUMAN	Q6N069	ENSG00000172766	
HPSE2_HUMAN	Q8WWQ2	ENSG00000172987	
RAPH1_HUMAN	Q70E73	ENSG00000173166	
TS1R1_HUMAN	Q7RTX1	ENSG00000173662	
GPR64_HUMAN	Q8IZP9	ENSG00000173698	ENSMUSG00000031298
PIGG_HUMAN	Q5H8A4	ENSG00000174227	
GRIPE_HUMAN	Q6GYQ0	ENSG00000174373	
PELI3_HUMAN	Q8N2H9	ENSG00000174516	
RIMS2_HUMAN	Q9UQ26	ENSG00000176406	
YSK4_HUMAN	Q56UN5	ENSG00000176601	
NMDZ1_HUMAN	Q05586	ENSG00000176884	ENSMUSG00000026959
DEAF1_HUMAN	O75398	ENSG00000177030	
UROL1_HUMAN	Q5DID0	ENSG00000177398	ENSMUSG00000054134

CD28_HUMAN	P10747	ENSG00000178562	
LX15B_HUMAN	O15296	ENSG00000179593	
TY3H_HUMAN	P07101	ENSG00000180176	
LAP4_HUMAN	Q14160	ENSG00000180900	ENSMUSG00000022568
EXOC7_HUMAN	Q9UPT5	ENSG00000182473	ENSMUSG00000020792
RGS6_HUMAN	P49758	ENSG00000182732	
RGS7_HUMAN	P49802	ENSG00000182901	ENSMUSG00000026527
CEP63_HUMAN	Q96MT8	ENSG00000182923	
NAC1_HUMAN	P32418	ENSG00000183023	ENSMUSG00000054640
BCOR_HUMAN	Q6W2J9	ENSG00000183337	ENSMUSG00000040363
EP400_HUMAN	Q96L91	ENSG00000183495	ENSMUSG00000029505
CHK2_HUMAN	O96017	ENSG00000183765	
AAT1_HUMAN	Q7Z4T9	ENSG00000183833	
SMOO_HUMAN	P53814	ENSG00000183963	ENSMUSG00000020439
WT1_HUMAN	P19544	ENSG00000184937	ENSMUSG00000016458
5NT1B_HUMAN	Q96P26	ENSG00000185013	
TF3B_HUMAN	Q92994	ENSG00000185024	
PRKN2_HUMAN	O60260	ENSG00000185345	
RA51D_HUMAN	O75771	ENSG00000185379	ENSMUSG00000018841
I28RA_HUMAN	Q8IU57	ENSG00000185436	
MUC1_HUMAN	P15941	ENSG00000185499	
LMBTL_HUMAN	Q9Y468	ENSG00000185513	
KCIP4_HUMAN	Q6PIL6	ENSG00000185774	ENSMUSG00000029088
IKAR_HUMAN	Q13422	ENSG00000185811	ENSMUSG00000018654
CLM1_HUMAN	Q8TDQ1	ENSG00000186074	
WWOX_HUMAN	Q9NZC7	ENSG00000186153	ENSMUSG00000004637
MKL2_HUMAN	Q9ULH7	ENSG00000186260	ENSMUSG00000009569
FCAR_HUMAN	P24071	ENSG00000186431	
MERL_HUMAN	P35240	ENSG00000186575	ENSMUSG00000009073
CEND2_HUMAN	Q96P48	ENSG00000186635	ENSMUSG00000032812
RHG08_HUMAN	Q9NSG0	ENSG00000186654	
HYAL3_HUMAN	O43820	ENSG00000186792	
TAU_HUMAN	P10636	ENSG00000186868	ENSMUSG00000018411
MITF_HUMAN	O75030	ENSG00000187098	ENSMUSG00000035158
NPSR1_HUMAN	Q6W5P4	ENSG00000187258	
P2RX2_HUMAN	Q9UBL9	ENSG00000187848	
DMBT1_HUMAN	Q9UGM3	ENSG00000187908	
CERKL_HUMAN	Q49MI3	ENSG00000188452	
FUSIP_HUMAN	O75494	ENSG00000188529	ENSMUSG00000028676
CLN3_HUMAN	Q13286	ENSG00000188603	ENSMUSG00000030720
RHCE_HUMAN	P18577	ENSG00000188672	
KI2L4_HUMAN	Q99706	ENSG00000189013	

KI2L4_HUMAN	Q99706	ENSG00000189013	
NCTR1_HUMAN	O76036	ENSG00000189430	
MGR7_HUMAN	Q14831	ENSG00000196277	
ZN124_HUMAN	Q15973	ENSG00000196418	
PDLI7_HUMAN	Q9NR12	ENSG00000196923	ENSMUSG00000021493
ACCN3_HUMAN	Q9UHC3	ENSG00000197150	ENSMUSG00000028973
RED1_HUMAN	P78563	ENSG00000197381	ENSMUSG00000020262
MIB2_HUMAN	Q96AX9	ENSG00000197530	
VP13A_HUMAN	Q96RL7	ENSG00000197969	
MBP_HUMAN	P02686	ENSG00000197971	ENSMUSG00000041607
MIER1_HUMAN	Q8N108	ENSG00000198160	ENSMUSG00000028522
CSF2R_HUMAN	P15509	ENSG00000198223	
ITSN2_HUMAN	Q9NZM3	ENSG00000198399	
CTND1_HUMAN	O60716	ENSG00000198561	ENSMUSG00000034101
DMD_HUMAN	P11532	ENSG00000198947	ENSMUSG00000045103
I5P2_HUMAN	P32019	ENSG00000204084	
SYT15_HUMAN	Q9BQS2	ENSG00000204176	
COBA2_HUMAN	P13942	ENSG00000204248	ENSMUSG00000024330
MSH5_HUMAN	O43196	ENSG00000204410	
NCTR3_HUMAN	O14931	ENSG00000204475	
LST1_HUMAN	O00453	ENSG00000204482	
GABR1_HUMAN	Q9UBS5	ENSG00000204681	
ATX2_HUMAN	Q99700	ENSG00000204842	
CKLF_HUMAN	Q9UBR5	ENSG00000205299	ENSMUSG00000054400
ITSN1_HUMAN	Q15811	ENSG00000205726	
MOG_HUMAN	Q16653	ENSG00000206456	
COLQ_HUMAN	Q9Y215	ENSG00000206561	
