# Supplementary material for: Comparative Component Analysis of Exons with Different Splicing Frequencies
Source: PLoS One. 2009 Apr 30;4(4):e5387. doi: 10.1371/journal.pone.0005387 (PMC2671145; doi:10.1371/journal.pone.0005387)
Supplement: Table S4 — Alternatively spliced human genes and orthologous NAS mouse genes (0.03 MB PDF) [file pone.0005387.s004.pdf]

Table S4. Alternatively spliced human genes and orthologous NAS mouse genes.

Species			
Alternatively spliced human genes			NAS Mouse genes
**SwissProt Name**	**SwissProt(acc)**	**Human Ensembl_Gene ID**	**Mouse Ensembl_Gene ID**
TCGAP_HUMAN	O14559	ENSG00000004777	ENSMUSG00000036882
BID_HUMAN	P55957	ENSG00000015475	ENSMUSG00000004446
CP343_HUMAN	Q9HB55	ENSG00000021461	ENSMUSG00000029727
CP343_HUMAN	Q9HB55	ENSG00000021461	ENSMUSG00000038656
CP343_HUMAN	Q9HB55	ENSG00000021461	ENSMUSG00000054417
CP343_HUMAN	Q9HB55	ENSG00000021461	ENSMUSG00000056035
CP343_HUMAN	Q9HB55	ENSG00000021461	ENSMUSG00000075551
CP343_HUMAN	Q9HB55	ENSG00000021461	ENSMUSG00000075552
BRD9_HUMAN	Q9H8M2	ENSG00000028310	ENSMUSG00000057649
F120A_HUMAN	Q9NZB2	ENSG00000048828	ENSMUSG00000038014
ELN_HUMAN	P15502	ENSG00000049540	ENSMUSG00000029675
LZTS1_HUMAN	Q9Y250	ENSG00000061337	ENSMUSG00000036306
AP3D1_HUMAN	O14617	ENSG00000065000	ENSMUSG00000020198
AFF4_HUMAN	Q9UHB7	ENSG00000072364	ENSMUSG00000049470
GLI2_HUMAN	P10070	ENSG00000074047	ENSMUSG00000048402
DPP8_HUMAN	Q6V1X1	ENSG00000074603	ENSMUSG00000032393
SIRT6_HUMAN	Q8N6T7	ENSG00000077463	ENSMUSG00000034748
ZCPW1_HUMAN	Q9H0M4	ENSG00000078487	ENSMUSG00000037108
PPE1_HUMAN	O14829	ENSG00000086717	ENSMUSG00000062168
BAXA_HUMAN	Q07812	ENSG00000087088	ENSMUSG00000003873
BAXB_HUMAN	Q07814	ENSG00000087088	ENSMUSG00000003873
BAXC_HUMAN	Q07815	ENSG00000087088	ENSMUSG00000003873
BAXD_HUMAN	P55269	ENSG00000087088	ENSMUSG00000003873
CT012_HUMAN	Q9NVP4	ENSG00000089091	ENSMUSG00000037259
NALP1_HUMAN	Q9C000	ENSG00000091592	ENSMUSG00000040575
HRH3_HUMAN	Q9Y5N1	ENSG00000101180	ENSMUSG00000039059
WFDC2_HUMAN	Q14508	ENSG00000101443	ENSMUSG00000000983
WFDC2_HUMAN	Q14508	ENSG00000101443	ENSMUSG00000069792
CM018_HUMAN	Q9H714	ENSG00000102445	ENSMUSG00000034959
RASF4_HUMAN	Q9H2L5	ENSG00000107551	ENSMUSG00000042129
PK2L1_HUMAN	Q9P0L9	ENSG00000107593	ENSMUSG00000037578
TRDMT_HUMAN	O14717	ENSG00000107614	ENSMUSG00000026723
SCNNA_HUMAN	P37088	ENSG00000111319	ENSMUSG00000030340
OGG1_HUMAN	O15527	ENSG00000114026	ENSMUSG00000030271
PERT_HUMAN	P07202	ENSG00000115705	ENSMUSG00000020673
UCHL5_HUMAN	Q9Y5K5	ENSG00000116750	ENSMUSG00000018189
TNI3K_HUMAN	Q59H18	ENSG00000116783	ENSMUSG00000040086
PTBP2_HUMAN	Q9UKA9	ENSG00000117569	ENSMUSG00000028134

ZO2_HUMAN	Q9UDY2	ENSG00000119139	ENSMUSG00000024812
TECT3_HUMAN	Q6NUS6	ENSG00000119977	ENSMUSG00000025008
APAF_HUMAN	O14727	ENSG00000120868	ENSMUSG00000019979
ADA1A_HUMAN	P35348	ENSG00000120907	ENSMUSG00000045875
PDLI2_HUMAN	Q96JY6	ENSG00000120913	ENSMUSG00000022090
OBP2A_HUMAN	Q9NY56	ENSG00000122136	ENSMUSG00000026919
OBP2A_HUMAN	Q9NY56	ENSG00000122136	ENSMUSG00000026936
OBP2A_HUMAN	Q9NY56	ENSG00000122136	ENSMUSG00000062061
CD244_HUMAN	Q9BZW8	ENSG00000122223	ENSMUSG00000004709
NCOA3_HUMAN	Q9Y6Q9	ENSG00000124151	ENSMUSG00000079640
WRIP1_HUMAN	Q96S55	ENSG00000124535	ENSMUSG00000021400
MOS1A_HUMAN	O14940	ENSG00000124615	ENSMUSG00000064120
MOS1B_HUMAN	Q9NZB8	ENSG00000124615	ENSMUSG00000064120
PCID2_HUMAN	Q5JVF3	ENSG00000126226	ENSMUSG00000038542
KIRR2_HUMAN	Q6UWL6	ENSG00000126259	ENSMUSG00000036915
IBA2_HUMAN	Q9BQI0	ENSG00000126878	ENSMUSG00000001864
BEST3_HUMAN	Q8N1M1	ENSG00000127325	ENSMUSG00000020169
RAB3I_HUMAN	Q96QF0	ENSG00000127328	ENSMUSG00000064181
AFAD_HUMAN	P55196	ENSG00000130396	ENSMUSG00000068036
ICB1_HUMAN	Q5TEJ8	ENSG00000130775	ENSMUSG00000037731
FCRLA_HUMAN	Q7L513	ENSG00000132185	ENSMUSG00000038421
TRIM5_HUMAN	Q9C035	ENSG00000132256	ENSMUSG00000078616
PPHLN_HUMAN	Q8NEY8	ENSG00000134283	ENSMUSG00000050528
PPHLN_HUMAN	Q8NEY8	ENSG00000134283	ENSMUSG00000079737
ASPX_HUMAN	P26436	ENSG00000134940	ENSMUSG00000032110
MTO1_HUMAN	Q9Y2Z2	ENSG00000135297	ENSMUSG00000032342
SP110_HUMAN	Q9HB58	ENSG00000135899	ENSMUSG00000079808
FLNB_HUMAN	O75369	ENSG00000136068	ENSMUSG00000025278
DCTN3_HUMAN	O75935	ENSG00000137100	ENSMUSG00000028447
TJAP1_HUMAN	Q5JTD0	ENSG00000137221	ENSMUSG00000012296
CASP1_HUMAN	P29466	ENSG00000137752	ENSMUSG00000025888
CUZD1_HUMAN	Q86UP6	ENSG00000138161	ENSMUSG00000040205
FRAS1_HUMAN	Q86XX4	ENSG00000138759	ENSMUSG00000034687
RGS3_HUMAN	P49796	ENSG00000138835	ENSMUSG00000048600
CAC1A_HUMAN	O00555	ENSG00000141837	ENSMUSG00000034656
CAN10_HUMAN	Q9HC96	ENSG00000142330	ENSMUSG00000026270
PAIRB_HUMAN	Q8NC51	ENSG00000142864	ENSMUSG00000036371
SYT14_HUMAN	Q8NB59	ENSG00000143469	ENSMUSG00000016200
VASH2_HUMAN	Q86V25	ENSG00000143494	ENSMUSG00000037568
HNRLL_HUMAN	Q8WVV9	ENSG00000143889	ENSMUSG00000024095
TRI55_HUMAN	Q9BYV6	ENSG00000147573	ENSMUSG00000060913
CBWD1_HUMAN	Q9BRT8	ENSG00000147996	ENSMUSG00000024878

RRFM_HUMAN	Q96E11	ENSG00000148187	ENSMUSG00000026887
ACF_HUMAN	Q9NQ94	ENSG00000148584	ENSMUSG00000052595
F10C1_HUMAN	Q70Z53	ENSG00000148690	ENSMUSG00000054237
NPAS3_HUMAN	Q8IXF0	ENSG00000151322	ENSMUSG00000021010
RAD17_HUMAN	O75943	ENSG00000152942	ENSMUSG00000021635
ING1_HUMAN	Q9UK53	ENSG00000153487	ENSMUSG00000045969
MMS19_HUMAN	Q96T76	ENSG00000155229	ENSMUSG00000025159
AFF2_HUMAN	P51816	ENSG00000155966	ENSMUSG00000031189
SMG1_HUMAN	Q96Q15	ENSG00000157106	ENSMUSG00000030655
MPIP3_HUMAN	P30307	ENSG00000158402	ENSMUSG00000044201
KAT_HUMAN	Q8NFU3	ENSG00000158769	ENSMUSG00000038235
GCFC_HUMAN	Q9Y5B6	ENSG00000159086	ENSMUSG00000022974
ABCG1_HUMAN	P45844	ENSG00000160179	ENSMUSG00000024030
PDE9A_HUMAN	O76083	ENSG00000160191	ENSMUSG00000041119
TOR2A_HUMAN	Q5JU69	ENSG00000160404	ENSMUSG00000009563
TOR2X_HUMAN	Q8N2E6	ENSG00000160404	ENSMUSG00000009563
PAQR6_HUMAN	Q6TCH4	ENSG00000160781	ENSMUSG00000078691
AIOL_HUMAN	Q9UKT9	ENSG00000161405	ENSMUSG00000018168
DISC1_HUMAN	Q9NRI5	ENSG00000162946	ENSMUSG00000079751
ATRIP_HUMAN	Q8WXE1	ENSG00000164053	ENSMUSG00000025646
TAGAP_HUMAN	Q8N103	ENSG00000164691	ENSMUSG00000033450
TAGAP_HUMAN	Q8N103	ENSG00000164691	ENSMUSG00000052031
TAGAP_HUMAN	Q8N103	ENSG00000164691	ENSMUSG00000079571
BAALC_HUMAN	Q8WXS3	ENSG00000164929	ENSMUSG00000022296
NOL6_HUMAN	Q9H6R4	ENSG00000165271	ENSMUSG00000028430
VDAC2_HUMAN	P45880	ENSG00000165637	ENSMUSG00000021771
NKX31_HUMAN	Q99801	ENSG00000167034	ENSMUSG00000022061
NALDL_HUMAN	Q9UQQ1	ENSG00000168060	ENSMUSG00000054999
ATX2L_HUMAN	Q8WWM7	ENSG00000168488	ENSMUSG00000032637
DFFB_HUMAN	O76075	ENSG00000169598	ENSMUSG00000029027
ZDH16_HUMAN	Q969W1	ENSG00000171307	ENSMUSG00000025157
CD8B_HUMAN	P10966	ENSG00000172116	ENSMUSG00000053044
NARGL_HUMAN	Q6N069	ENSG00000172766	ENSMUSG00000022020
HPSE2_HUMAN	Q8WWQ2	ENSG00000172987	ENSMUSG00000074852
TS1R1_HUMAN	Q7RTX1	ENSG00000173662	ENSMUSG00000028950
PIGG_HUMAN	Q5H8A4	ENSG00000174227	ENSMUSG00000029263
PELI3_HUMAN	Q8N2H9	ENSG00000174516	ENSMUSG00000024901
CD28_HUMAN	P10747	ENSG00000178562	ENSMUSG00000026012
CHK2_HUMAN	O96017	ENSG00000183765	ENSMUSG00000029521
5NT1B_HUMAN	Q96P26	ENSG00000185013	ENSMUSG00000020622
TF3B_HUMAN	Q92994	ENSG00000185024	ENSMUSG00000011158
I28RA_HUMAN	Q8IU57	ENSG00000185436	ENSMUSG00000062157

MUC1_HUMAN	P15941	ENSG00000185499	ENSMUSG00000042784
HYAL3_HUMAN	O43820	ENSG00000186792	ENSMUSG00000036091
NPSR1_HUMAN	Q6W5P4	ENSG00000187258	ENSMUSG00000043659
CERKL_HUMAN	Q49MI3	ENSG00000188452	ENSMUSG00000075256
RHCE_HUMAN	P18577	ENSG00000188672	ENSMUSG00000028825
NCTR1_HUMAN	O76036	ENSG00000189430	ENSMUSG00000062524
MGR7_HUMAN	Q14831	ENSG00000196277	ENSMUSG00000056755
CSF2R_HUMAN	P15509	ENSG00000198223	ENSMUSG00000059326
SYT15_HUMAN	Q9BQS2	ENSG00000204176	ENSMUSG00000041479
