# Supplementary material for: Comparative Component Analysis of Exons with Different Splicing Frequencies
Source: PLoS One. 2009 Apr 30;4(4):e5387. doi: 10.1371/journal.pone.0005387 (PMC2671145; doi:10.1371/journal.pone.0005387)
Supplement: Table S5 — The proportions of each exon groups for each studied splicing pattern in alternatively spliced human and mouse genes (0.02 MB PDF) [file pone.0005387.s005.pdf]

Table S5. The proportions of each exon groups for each studied splicing pattern in alternatively spliced human and mouse genes.

Splicing pattern	Human exon groups				Mouse exon groups			
	G1	G2	G3	G4	G1	G2	G3	G4
Cassette exons	0.1348	0.1422	0.1421	0.1374	0.1579	0.1503	0.1613	0.1546
Mutually exclusively exons	0.0293	0.0308	0.0233	0.0330	0.0474	0.0327	0.0387	0.0435
Retained intron	0.0469	0.0474	0.0413	0.0302	0.0368	0.0458	0.0258	0.0338
Alternative acceptor site	0.0567	0.0592	0.0594	0.0495	0.0421	0.0458	0.0323	0.0435
Alternative donor site	0.0508	0.0498	0.0517	0.0357	0.0316	0.0327	0.0258	0.0290
