# Supplementary material for: Comparative Component Analysis of Exons with Different Splicing Frequencies
Source: PLoS One. 2009 Apr 30;4(4):e5387. doi: 10.1371/journal.pone.0005387 (PMC2671145; doi:10.1371/journal.pone.0005387)
Supplement: Table S6 — Regulatory elements as divided into 6 main categories based on their biological functions (0.02 MB PDF) [file pone.0005387.s006.pdf]

Table S6. Regulatory elements as divided into 6 main categories based on their biological functions.

ESE Category		
**ESE_1**	SF2/ASF	SF2/ASF(IgM-BRCA1)
**ESE_2**	SC35	
**ESE_3**	SRp40	SRp55
**ESE_4**	BranchSite	
**ESE_5**	3’ss_U2_human	3’ss_U2_mouse
**ESE_6**	5’ss_U2_human	5’ss_U2_mouse
