# Supplementary material for: Comparative Component Analysis of Exons with Different Splicing Frequencies
Source: PLoS One. 2009 Apr 30;4(4):e5387. doi: 10.1371/journal.pone.0005387 (PMC2671145; doi:10.1371/journal.pone.0005387)
Supplement: Table S7 — Numbers of repetitive elements in different groups of NAS mouse exons and orthologous human exons (0.02 MB PDF) [file pone.0005387.s007.pdf]

Table S7. Numbers of repetitive elements in different groups of NAS mouse exons and orthologous human exons.

Exon group	NAS mouse	human G1	human G2	human G3	human G4
**Repeat element types**					
**DNA type**					
DNA	0	2	0	0	0
DNA/AcHobo	0	3	0	0	0
DNA/Mariner	0	1	0	0	0
DNA/MER1_type	6	27	3	0	0
DNA/MER2_type	0	7	0	0	0
DNA/Tc2	0	1	0	0	0
DNA/TcMar?	0	1	0	0	0
**LINE type**					
LINE/CR1	0	1	0	0	0
LINE/L1	5	10	2	0	0
LINE/L2	4	23	0	1	0
LINE/RTE	0	4	0	0	0
**LTR type**					
LTR/ERV1	0	6	0	0	0
LTR/ERVK	1	3	0	0	0
LTR/ERVL	3	0	0	0	0
LTR/MaLR	1	13	0	0	0
**SINE Type**					
SINE/Alu	27	56	3	4	1
SINE/MIR	5	30	2	1	0
SINE/tRNA	0	2	0	0	0
SINE/B2	7	0	0	0	0
SINE/B4	8	0	0	0	0
SINE/ID	4	0	0	0	0
**Other type**					
Low_complexity	47	60	9	14	8
Simple_repeat	46	52	5	8	2
